# Resveratrol Brain Delivery for Neurological Disorders Prevention and Treatment

**DOI:** 10.3389/fphar.2018.01261

**Published:** 2018-11-20

**Authors:** Stephanie Andrade, Maria João Ramalho, Maria do Carmo Pereira, Joana A. Loureiro

**Affiliations:** LEPABE, Department of Chemical Engineering, Faculty of Engineering of the University of Porto, Porto, Portugal

**Keywords:** resveratrol, nanoparticles, neurological disorders, encapsulation, brain delivery

## Abstract

Resveratrol (RES) is a natural polyphenolic non-flavonoid compound present in grapes, mulberries, peanuts, rhubarb and in several other plants. Numerous health effects have been related with its intake, such as anti-carcinogenic, anti-inflammatory and brain protective effects. The neuroprotective effects of RES in neurological diseases, such as Alzheimer’s (AD) and Parkinson’s (PD) diseases, are related to the protection of neurons against oxidative damage and toxicity, and to the prevention of apoptotic neuronal death. In brain cancer, RES induces cell apoptotic death and inhibits angiogenesis and tumor invasion. Despite its great potential as therapeutic agent for the treatment of several diseases, RES exhibits some limitations. It has poor water solubility and it is chemically instable, being degraded by isomerization once exposed to high temperatures, pH changes, UV light, or certain types of enzymes. Thus, RES has low bioavailability, limiting its biological and pharmacological benefits. To overcome these limitations, RES can be delivered by nanocarriers. This field of nanomedicine studies how the drug administration, pharmacokinetics, and pharmacodynamics are affected by the use of nanosized materials. The role of nanotechnology, in the prevention and treatment of neurological diseases, arises from the necessity to mask the physicochemical properties of therapeutic drugs to prolong the half-life and to be able to cross the blood–brain barrier (BBB). This can be achieved by encapsulating the drug in a nanoparticle (NP), which can be made of different kinds of materials. An increasing trend to encapsulate and direct RES to the brain has been observed. RES has been encapsulated in many different types of nanosystems, as liposomes, lipid and polymeric NPs. Furthermore, some of these nanocarriers have been modified with targeting molecules able to recognize the brain areas. Then, this article aims to overview the RES benefits and limitations in the treatment of neurological diseases, as the different nanotechnology strategies to overcome these limitations.

## Introduction

**FIGURE 1:**
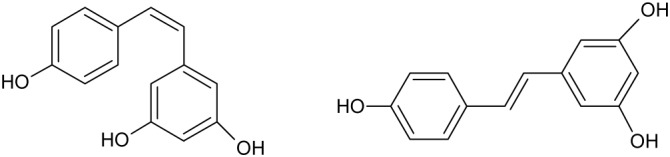
Chemical structure of *cis*- and *trans*- resveratrol.

Resveratrol (RES) is a natural non-flavonoid polyphenolic molecule found in several sources, such as fruits (i.e., grapes, mulberries, and peanuts), roots, grains, seeds, flowers, vegetables, and tea (i.e., rhubarb, green tea, and black tea) and wine (Berman et al., 2017). RES exhibits two geometrical isomers, *cis*- and *trans*-RES, being the last the biologically active form (Figure 1) (Wang and Chatterjee, 2017). *Trans*-RES can be converted into the *cis*-form after exposure to UV radiation (Yang et al., 2015).

Numerous therapeutic effects have been associated with RES administration such as antioxidant, anti-inflammatory, anticarcinogenic, cardioprotective, and analgesic properties (Rauf et al., 2017). Thus, RES has been studied as therapeutic agent for cardiovascular disease, obesity, diabetes, and recently with an increasing relevance for the neurological disorders, including brain tumors (gliomas), Alzheimer’s (AD) and Parkinson (PD) diseases (Tellone et al., 2015; Martin, 2017; Rauf et al., 2018).

Despite its huge potential as a therapeutic agent, the administration of RES faces several limitations. RES exhibits low bioavailability due to its short biological half-life, its rapid metabolism and clearance, and being chemically instable, highly photosensitive and poorly soluble in water (Francioso et al., 2014; Wang et al., 2017). Its low bioavailability hampers drug accumulation at necessary concentrations for successful therapy in target tissues (Berman et al., 2017). RES is quickly absorbed after reaching the bloodstream: it is metabolized in the liver and rapidly eliminated. Then, since the success of a neurological disorder therapy depends on a great extent on drug bioavailability, new strategies are required (Ghersi-Egea et al., 2009).

A growing effort has been applied to overcome these issues and to find novel approaches for the treatment of neurological diseases with RES. Nanotechnology have been suggested as a suitable strategy for RES delivery to enhance its therapeutic activity (Summerlin et al., 2015). Through nanoencapsulation is possible to enhance RES bioavailability by increasing its solubility in water and preventing degradation. Nanoencapsulation will also allow decreasing RES toxicity in healthy tissues (Amri et al., 2012; Arora and Jaglan, 2017). In the last decade, several nanomaterials have been proposed as drug delivery systems (DDSs) (Peres et al., 2012; Khan et al., 2015; Ramalho et al., 2015a,b; Ramalho and Pereira, 2016). Each nanomaterial exhibits different biochemical and physicochemical properties and depending on the drug to be encapsulated and the disease to be treated, the most suitable nanomaterial should be selected based on its characteristics. In addition, biological barriers must be considered during the design and development of the chosen nanocarrier (Blanco et al., 2015).

Among the existent biological barriers, the blood–brain barrier (BBB) is one of the main drawbacks for brain delivery. This barrier consists on a monolayer of capillary endothelial cells that block the path of over 98% of small-molecule drugs and 100% of large-molecule (>500 Da) (Cardoso et al., 2010), protecting the brain microenvironment from toxic substances present in the bloodstream, but allowing the transport of nutrients (Groothuis, 2000; Liebner et al., 2011). Thus, BBB hinders the development of novel therapies for brain disorders (Pardridge, 2009; Loureiro et al., 2014b; Bhowmik et al., 2015).

The capability of nanosystems to cross the BBB depends on their physicochemical properties, and therefore nanocarriers must meet several requirements as being non-toxic, able to carry the desired drug and able to interact with receptors present at the BBB (Chen and Liu, 2012; Masserini, 2013). Some authors were able to develop efficient nanosystems for the encapsulation of RES and its delivery to the brain tissue (Hao et al., 2016; Ethemoglu et al., 2017; Trotta et al., 2018). Several studies prove that modification of the surface of the nanosystem can enhance the transport across this barrier (Loureiro et al., 2013a, 2015a; Neves et al., 2016; Shen et al., 2018). The modifications strategies can be based on attaching to the nanomaterial surface different moieties that improve the recognition of the nanoparticle (NP) by the BBB receptors (Loureiro et al., 2015b, 2016; Li et al., 2017).

As mentioned above, a growing number of studies have focused on the treatment of several neurological diseases with RES encapsulated in NPs. This review will focus on RES therapeutic mechanisms in the prevention and treatment of glioma tumors, AD and PD, and nanotechnology-based strategies for its use in these diseases.

## Alzheimer’s Disease

The incidence of AD is related with the age. Affecting around 35 million of people in the world, AD is the most common form of dementia (Brookmeyer et al., 2007). This disease is progressive and induces memory injury and language, cognitive deficiency and behavior modifications. The neuropathological characteristics of AD are the extracellular senile plaques and neurofibrillary tangles (NFTs), composed by deposits of amyloid-beta peptide (Aβ) and by hyperphosphorylation and abnormal deposition of tau protein, respectively (Selkoe, 2001; Klafki et al., 2006). The Aβ aggregation induces the formation of insoluble fibrils, which accumulate in senile plaques (Clippingdale et al., 2001; Selkoe, 2001; Klafki et al., 2006; Rocha et al., 2012; Loureiro et al., 2013b). This process results from the imbalance between Aβ production and its clearance, that plays a crucial function in the AD progress (Hardy and Selkoe, 2002; Ahn et al., 2007). Insoluble fibrillar protein aggregates are considered a pathological feature of AD. Several tools have been studied to prevent and treat this disease (Loureiro et al., 2012, 2014a; Gomes et al., 2015; Carneiro et al., 2017). Therefore, it is indispensable to find drugs that prevent or inhibit the Aβ aggregation (Ahn et al., 2007; Ramalho et al., 2015b). Thus, RES has aroused a great interest in the scientific community to prevent and treat the AD, as will be discussed below.

### Resveratrol in Alzheimer’s Disease

**FIGURE 2:**
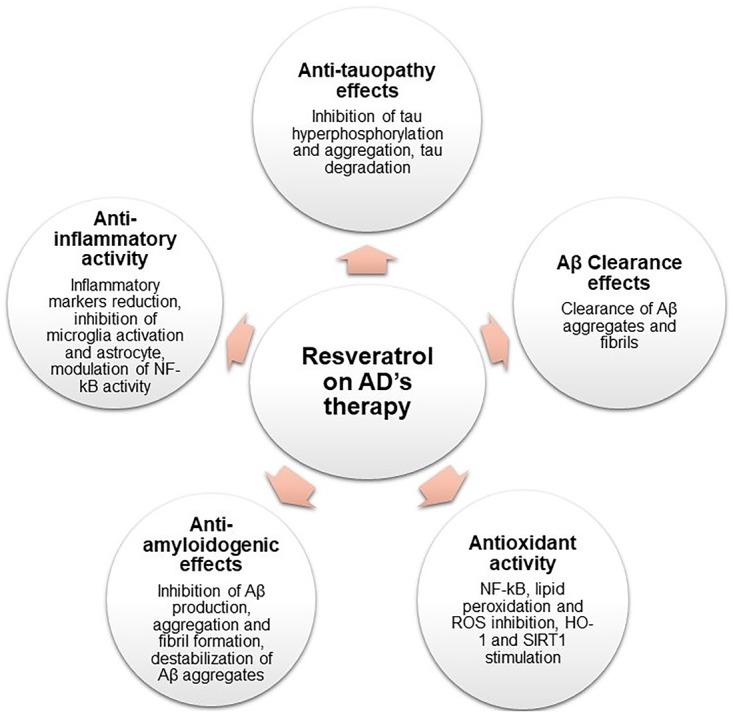
Schematic representation of RES pharmacological activities on AD therapy.

The beneficial effects of RES in AD have been studied in recent years, in *in vitro* as well as *in vivo* assays. This section describes the several mechanisms of action of RES on AD as presented in Figure 2.

#### Antioxidant Activity

Oxidative stress has been related with AD pathogenesis. Some products of oxidative stress, such as reactive oxygen species (ROS), interact with proteins, nucleic acids, and membrane polyunsaturated fatty acids. This causes lipid oxidation, decrease of membrane integrity and augmented permeability to Ca^2+^ in plasma membrane (Abolfathi et al., 2012; Li et al., 2012), which in turn favors the connection between nuclear factor kappa B (NF-κB) and specific regions of DNA, that promotes cell and tissue injuries leading to cell death (Draczynska-Lusiak et al., 1998; Ahmed et al., 2017).

The therapeutic effects of RES are related to its antioxidant activity (Rege et al., 2014; Ahmed et al., 2017). Some authors observed that RES has protective activity in PC12 cells through the prevention of NF-κB activation, inhibiting the apoptosis (Jang and Surh, 2003; Seo et al., 2018). Additionally, RES decreased the lipid peroxide levels in H19-7 cells exposed to Aβ (Rege et al., 2015), inhibited membrane lipid peroxidation and decreased the toxic effects produced by ROS (Vingtdeux et al., 2008; Rege et al., 2015).

Tamagno et al. (2002) concluded that ROS production promotes the activation of β-secretase, stimulating the Aβ production (Murakami et al., 2005). In addition, Aβ-mitochondrial interactions induce oxidative stress and mitochondrial damages (Anandatheerthavarada et al., 2003). Also, ROS production results in oxidative damage to mitochondria causing the reduction of mitochondrial energy production (Reddy and Beal, 2005). Thus, creating a vicious cycle, where ROS promote Aβ production, which in turn, promotes ROS production (Anekonda, 2006; Li et al., 2012). In PC12 cells, RES decreased the modifications observed in mitochondrial membranes through the inhibition of ROS accumulation (Jang and Surh, 2003; Wang et al., 2018).

The free radical production is related with the cognitive impairment observed in AD. Studies proved that RES could ameliorate cognitive impairment, increasing glutathione levels, an intracellular free radical scavenger, improving the cell viability (Savaskan et al., 2003; Kumar et al., 2007). Also, RES showed to reduce the high malondialdehyde and nitrite levels observed in AD rats (Kumar et al., 2007; Al-Bishri et al., 2017). Additionally, *in vitro* evidences showed that RES improved the functional recovery and reduced the DNA fragmentation and apoptosis through the modulation of glutamate uptake activity and S100B secretion (Kutuk et al., 2004; de Almeida et al., 2007). Additionally, RES inhibits the expression of proteins that lead to oxidative stress, like glycogen synthase kinase 3 (GSK-3β), as well inhibits intracellular enzymes including cyclooxygenase, lipoxygenases, or nitric oxide synthase (Bastianetto et al., 2000).

Some studies have demonstrated that an endogenous enzyme, known as heme oxygenase (HO-1), has neuroprotective properties, providing resistance against oxidative stress (Bastianetto and Quirion, 2010). RES was described as a stimulator of HO-1 activity, protecting AD models against oxidative stress (Kwon et al., 2011; Li et al., 2012).

Still, sirtuin 1 (SIRT1) plays an important role against the oxidative stress, protecting the neurons. An *in vitro* study suggested that RES protected SK-N-BE cells against the oxidative stress and toxicity produced by Aβ, through the SIRT1 activation that inhibited the hydrogen peroxide production (Albani et al., 2009).

#### Anti-amyloidogenic Effects

Aβ monomers with α-helix conformation are produced by the sequential action of β- and γ-secretases responsible for the proteolytic cleavage of amyloid precursor protein. Then, a transition in the conformation from α-helix to β-sheet is observed. Aβ monomers, in particular Aβ_1–42_, rapidly form self-aggregates to produce oligomer aggregates that are known to initiate the pathogenic cascade. Posteriorly, the aggregation of oligomers forms protofibrils, which in turn form fibrils and then Aβ plaques (Walsh and Selkoe, 2007; Ahmed et al., 2010). Initially, it was assumed that only Aβ fibrils could exert toxic effects. However, in recent years studies have revealed that Aβ oligomers can be more toxic than mature fibrils by inducing synaptic dysfunction, playing a key role in the development of the disease (Walsh and Selkoe, 2007; Arendt, 2009; Ballard et al., 2011). Thus, small-molecule drugs, which interfere with the Aβ–aggregation inhibiting the formation of the supposed toxic Aβ oligomers, are a suitable approach in the prevention and treatment of AD (Klafki et al., 2006).

**FIGURE 3:**
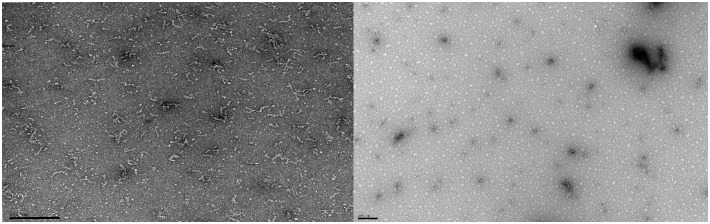
Transmission electron microscopy analysis of the resveratrol effect on Aβ_1–42_ aggregation. The Aβ_1–42_ concentration was 25 μM and the resveratrol concentration was 80 μM. The samples were incubated at 37°C in phosphate buffered saline buffer. The left side of the figure represents the incubation of Aβ_1–42_ without RES, and the right side shows Aβ_1–42_ incubated with RES. As shown in the figure RES prevented the formation of amyloid fibrils. The scale bar corresponds to 200 nm.

Evidences showed that RES delays or even inhibits the Aβ aggregation (Feng et al., 2009; Andrade et al., 2015; Loureiro et al., 2017). As observed in Figure 3 the formation of amyloid fibrils was prevented with RES treatment, through the binding of RES to the N-terminus of Aβ monomers (Fu et al., 2014). RES altered the Aβ_1–42_ oligomers conformation, prevented the oligomers aggregation, the Aβ_1–42_ fibril formation and destabilized Aβ aggregates (Feng et al., 2009; Mishra et al., 2009; Pasinetti et al., 2015).

Additionally, RES has the ability to interfere in aggregation indirectly. The aggregation of Aβ plaques can be prevented by the binding of a transporter protein called transthyretin. A study showed that RES increased the binding of transthyretin to Aβ oligomers (Ribeiro et al., 2012).

Also, RES reduced the Aβ production (Pasinetti et al., 2015) through the activation of SIRT1 that activate the α-secretase (Donmez et al., 2010; Granzotto and Zatta, 2014) and the reduction of the activity of β-secretase (Jeon et al., 2007; Porquet et al., 2014). Additionally, and since high levels of cholesterol promote the Aβ production, the ability of RES to maintain the cholesterol homeostasis proved to be determinant (Ahmed et al., 2017).

In a *in vivo* study, RES inhibited the formation of amyloid plaques in medial cortex, striatum and hypothalamus, in 48, 89, and 90%, respectively (Karuppagounder et al., 2009) and decreased the insoluble Aβ_1–42_ level in hippocampus (Zhao et al., 2015). Also, RES converted toxic species such as oligomers, protofibrils, and fibrils into non-toxic aggregated (Ladiwala et al., 2010; Rushworth et al., 2013). Concerning the non-toxic species like Aβ monomers, RES did not induce alterations (Ladiwala et al., 2010). Another study proved that when RES was incubated with Aβ, it has capacity to decrease the length and the amount of fibrils (Ge et al., 2012) and amyloid plaques (Porquet et al., 2014). Evidences suggested that RES markedly slowed down the formation of Aβ fibrils and their extension by disrupting Aβ_1–42_ hydrogen bonding. Also, an *in vitro* experiment showed that RES reduced the Aβ secretion and Aβ fibrils accumulation (Marambaud et al., 2005).

A study, using neuronal and non-neuronal cells and mice models, showed that the anti-amyloidogenic activity of RES is mediated by AMP-activated protein kinase (AMPK). RES stimulated the AMPK activation (Vingtdeux et al., 2010; Porquet et al., 2013), which leads to the decrease of Aβ levels and extracellular Aβ accumulation (Vingtdeux et al., 2010).

#### Aβ Clearance Effects

The imbalance between Aβ production and the elimination of the peptide leads to the progress of AD (Kurz and Perneczky, 2011). The augmented levels of Aβ peptide found in the brain are a consequence of the overproduction and/or the reduced clearance of Aβ (Bates et al., 2009). Some causes are pointed for the deficient Aβ elimination including the increase of aggregation and difficulty to remove Aβ through the BBB (Mawuenyega et al., 2010; Sagare et al., 2012). Thus, this mechanism has been studied over the past several years (Tanzi et al., 2004; Kurz and Perneczky, 2011).

Resveratrol promotes intracellular Aβ clearance of aggregates (Marambaud et al., 2005; Pasinetti et al., 2015) and preformed Aβ_1–42_ fibrils *in vitro* (Feng et al., 2009) through promotion of intracellular proteasomal activity (Marambaud et al., 2005; Bastianetto et al., 2015). Besides that, RES stimulated the activation of AMPK target mTOR, a mammalian target of rapamycin that regulates the cellular energy homeostasis, triggering the autophagy and lysosomal Aβ degradation (Vingtdeux et al., 2010, 2011).

Low-density lipoprotein receptor-related protein 1 and several Aβ-degrading enzymes were reported to be stimulators of Aβ clearance. Studies proved that RES activates these proteins, stimulating the Aβ clearance. As examples, the enzyme neprilysin seems to be responsible for the Aβ degradation. RES increased the neprilysin levels, decreasing the Aβ deposition and promoting Aβ degradation (El-Sayed and Bayan, 2015).

On the other hand, some works demonstrated that the BBB plays a key role in Aβ clearance and that its disintegration can lead to inefficient clearance (Deane et al., 2004). *In vivo* studies showed that RES protected the BBB integrity (Saha et al., 2012), through the increase of claudin-5 expression and decrease of the receptor for advanced glycation end products (RAGE) (Zhao et al., 2015; Moussa et al., 2017). RAGE is a receptor of the immunoglobulin super family that is found in BBB and that is responsible for the Aβ transport, from the blood to the brain, leading to mitochondrial dysfunction (Takuma et al., 2009). RAGE levels are found to be higher in AD pathology. A study proved that RES reduced the RAGE expression in vascular cells (Slevin et al., 2012).

#### Anti-tauopathy Effects

Tau, the major constituent of NFTs, is a microtubule-associated protein with the function of assembling and stabilizing microtubules in the neuronal cell and it is also involved in the axoplasmatic transport. These characteristic inclusions observed in AD neurons are constituted by hyperphosphorylated tau (Lee et al., 2001; Goedert et al., 2006). The amyloid cascade hypothesis proposes that high levels of Aβ induces modifications in tau protein with consequent formation of NFTs, which causes the loss of synapses and neurons in areas related with cognitive functions such as memory (Gotz et al., 2004; Small and Duff, 2008). Tau is a soluble protein, but when NFTs formation happens, insoluble aggregates are also formed, which leads to the disruption of the structure and function of neurons. In an early stage, tau monomers aggregate forming oligomers, which in turn aggregate into a β-sheet and form NFTs (Meraz-Ríos et al., 2010). Human trials revealed a direct relationship between the degree of the disease and the amount of NFTs (Wilcock and Esiri, 1982; Arriagada et al., 1992). Therefore, drugs with the ability to prevent the tau aggregation or block its hyperphosphorylation can be a potential treatment approach (Lee and Trojanowski, 2006; Schneider and Mandelkow, 2008).

Some studies showed the potential of RES against tauopathy related with AD, exhibiting a reduction of tau levels in rats (Al-Bishri et al., 2017; Sadhukhan et al., 2018) and inhibition of tau hyperphosphorylation and aggregation (Pasinetti et al., 2015; He et al., 2017). This reduction can be associated with the ability of RES to upregulate BAG2 levels, an endogenous protein responsible for removing the accumulated tau (Patil et al., 2013). Another study concluded that the ability of RES to protect against tau hyperphosphorylation and to stimulate the dephosphorylation of tau protein, is related with the inhibition of GSK-3β and CaMKII and the activation of protein phosphatase 2 (PP2A) (He et al., 2017; Jhang et al., 2017). PP2A is one of the main phosphatases that causes tau dephosphorylation. RES is a stimulator of PP2A activity reducing tau phosphorylation (Schweiger et al., 2017). Also, RES promoted the proteasomal degradation of tau through activating SIRT1 that induces the deacetylation of acetylated tau (Min et al., 2010).

#### Anti-inflammatory Effects

Neuroinflammation was described as a marker of AD pathology, playing a key role in AD progress, leading to the neurodegeneration and cognitive impairment (Granzotto and Zatta, 2014). *In vivo* and *in vitro* evidences suggest that Aβ aggregation stimulates neuroinflammation (Capiralla et al., 2012), as well as the insoluble Aβ deposits and NFTs (Wenk, 2003). Several studies proved that inflammatory responses, including the activation of macrophages, lymphocytes, astrocytes, and microglia lead to the expression of pro- and anti-inflammatory mediators like chemokines, neurotransmitters, cytokines, ROS, and complement factors (Heneka and O’Banion, 2007; Tansey et al., 2007), which promote the Aβ production (Ma et al., 2014), leading to neuroinflammation and even cell death (Liu and Hong, 2003).

Several studies described RES as a strong anti-inflammatory molecule (Chen et al., 2013; Moussa et al., 2017). There are several mechanisms associated with its anti-inflammatory effects (Granzotto and Zatta, 2014). Numerous studies showed that RES inhibited the secretion and activity of several cytokines and molecules with inflammatory activity (Lu et al., 2010; Zhang et al., 2010). Among these molecules are ROS, interleukins, nitric oxide (Kim et al., 2006; Huang et al., 2011), monocyte chemoattractant protein-1 and prostaglandin E2 (Kim et al., 2006). Also, an *in vivo* study revealed that RES decreased inflammatory markers such as IL-6, CRP, TNF-α, and TGF-β (Lu et al., 2010; Al-Bishri et al., 2017).

Numerous *in vitro* and *in vivo* works demonstrated that RES inhibited the activation of microglia (Zhang et al., 2010; Capiralla et al., 2012) and astrocyte (Scuderi et al., 2014), modulating the NF-κB activity (Zhao et al., 2015; Sathya et al., 2017) and inhibiting the TLR4/NF-κB/STAT signaling cascade (Capiralla et al., 2012). Also, RES activated the cAMPPRKA-AMPK-SI RT1 signaling pathway, that stimulated the autophagy, thus reducing the inflammation (Chen et al., 2013).

On the other hand, matrix metallopeptidase 9 (MMP9) was considered as displaying a key role in neuroinflammation. Studies showed that MMP9 actives several cytokines and chemokines, contributes to the BBB rupture and facilitates release of leukocytes in brain parenchyma. RES reduced MMP9 levels (Vafadari et al., 2016) and the input of leukocytes and others inflammatory mediators into the brain, and regulated the BBB permeability (Moussa et al., 2017).

SIRTs were also described as important for neurodegeneration, controlling several processes, including inflammation. Two categories of SIRTs, SIRT1 and SIRT2, exert an influence on neurons. While SIRT1 has neuroprotective activity, SIRT2 promotes neurodegeneration (Scuderi et al., 2014). Findings suggests that RES is capable of binding to the N-terminal of SIRT1 increasing their activity (Milne and Denu, 2008; Feng et al., 2013), decreasing Aβ accumulation (Kim et al., 2007; Feng et al., 2013), protecting against neurotoxicity (Porquet et al., 2013) and cell death (Sinclair and Guarente, 2014; Sathya et al., 2017).

### Nanocarriers for Resveratrol Delivery in Alzheimer’s Disease

As mentioned above, RES exhibits several beneficial properties for AD, which can be enhanced by the use of NPs. Thus, some authors proposed promising RES DDS to prevent and treat AD (Table 1). Frozza et al. (2010) prepared lipid-core nanocapsules (NCs) of capric/caprylic triglyceride and sorbitan monostearate, using the interfacial deposition of the polymer technique. The attained NCs showed an average size of 250 nm, with a negative zeta potential and an encapsulation efficiency of almost 100%. The NCs remained stable for 3 months at 25°C. This research aimed the study of the biodistribution of the developed NCs in the brain tissue of healthy rats. *In vivo* evidences suggested a higher concentration of encapsulated RES in the brain, kidney and liver tissues than for the free RES. Also, animals treated with encapsulated RES showed less gastrointestinal damage (Frozza et al., 2010).

**Table 1:** Currently developed DDS for RES delivery for AD’s therapy.

Carrier	Material	Coating	Targeting ligand	Development phase	Major findings	Reference
Lipid-core NCs	Capric/caprylic triglyceride and orbitan monostearate	No	No	*In vivo*	Improved biodistribution of RES in the brain, liver and kidney tissues. Reduction of harmful effects caused by Aβ with improvement of memory and learning.	Frozza et al., 2010, 2013b
					Neuroprotective effects against ROS formation and cell death.	
Polymeric micelles	PCL	PEG	No	*In vitro*	Protection of PC12 cells against Aβ-induced oxidative stress.	Lu et al., 2009
SLN	Cetyl palmitate	Polysorbate 80 and DSPE-PEG and LissRhod-PE	OX26	*In vitro*	Improvement of anti-aggregation effect of RES, ensuring the prevention of Aβ fibrillation.	Loureiro et al., 2017
					Improvement of the SLN transcytosis with OX26 functionalization.	

Later, the same group tested these NCs in rats exposed to Aβ, comparing the neuroprotective effects of RES-loaded NCs with free RES. The results showed that RES-loaded NCs decreased the harmful effects caused by Aβ, such as, memory loss, learning difficulty, reduced synaptophysin levels, activated astrocytes and microglial cells, among others. Free RES partially improved de adverse effects of Aβ. Improved efficiency of RES-loaded NCs is due to NCs’ ability to increase the RES concentration in brain tissue (Frozza et al., 2013a,b). Also, assays with rat organotypic hippocampal cultures showed that lipid-core NCs improved the neuroprotective effects of RES against ROS formation and cell death caused by Aβ, inhibiting the release of TNF-α, IL-1β, and IL-6. In addition, loaded-RES increased the release of IL-10 preventing or decreasing the glial cells and JNK activation (Frozza et al., 2013a). Thus, the encapsulation of RES in lipid-core NCs represents a promising approach in AD prevention and treatment.

Lu and colleagues developed and characterized RES-loaded polymeric micelles. The micelles were composed by poly(caprolactone) (PCL), an amphiphilic block co-polymer. The micelles exhibited a size less than 100 nm and a spherical shape with a smooth surface. The NPs were coated with polyethylene glycol (PEG). The DDS presented a high value of encapsulation efficiency. In this work, the group evaluated the effects of free and loaded-RES on PC12 cells viability. Free RES proved to be cytotoxic, not protecting against Aβ and decreasing cell viability. Instead, loaded-RES was non-toxic and protected PC12 cells against Aβ through the reduction of oxidative stress and caspase-3 activity. Therefore, this nanosystem seems to have a great potential as therapeutic method to treat AD (Lu et al., 2009).

Recently, Loureiro and co-workers encapsulated RES into solid lipid nanoparticles (SLN) composed by 1,2-distearoyl-sn-glycero-3-phosphoethanolamine-*N*-maleimide(polyethylene glycol)-2000 (DSPE-PEG) and 1,2-dipalmitoyl-sn-glycero-3-phosphoethanolamine-*N*-lissamine rhodamine B sulfonyl (LissRhod-PE). SLN were characterized through their size, zeta potential, polydispersity index, and morphology. SLN presented an average size of 176 nm, a neutral zeta potential and a polydispersity index of 0.16 (Loureiro et al., 2017). The SLN presented a high value of encapsulation efficiency, around 94%. The stability of the prepared nanosystems was assessed, and the results showed that SLN were stable for 3 months. SLN showed to improve the anti-aggregation effect of RES, ensuring the prevention of Aβ fibrillation. To direct the developed nanocarrier to the BBB, SLN were functionalized with OX26 monoclonal antibody molecules (mAb), a mAb that recognizes the transferrin receptor overexpressed in BBB. Also, the ability of OX26 for brain targeting was compared conjugating LB 509 mAb, an antibody, which recognize α-synuclein, a protein non-specific to the BBB. The stability of both formulations was tested, and results showed that SLNs conjugated with mAb were stable for 1 month. An *in vitro* BBB model was used to evaluate SLN brain targeting potential. Results showed the transport of SLN through the BBB model was twofold higher for the OX26-modifed SLN than for SLNs functionalized with LB 509, and fourfold higher when compared with unfunctionalized SLNs. Consequently, the transcytosis of SLN was improved with OX26 functionalization (Loureiro et al., 2017). Thus, SLN conjugated with OX26 represent a promising nanosystem to the brain delivery of RES.

## Parkinson’s Disease

After AD, PD is the most common neurodegenerative disorder that causes motor impairment on the middle-aged or elderly population (Schapira, 2009). The symptoms include irregular onset of bradykinesia, disturbance in gait, rigidity, and resting tremor (Schapira et al., 2006; Wang et al., 2011; Chopra et al., 2013).

A pathological mark of PD is the decrease of nigral dopaminergic neurons that are responsible for the dopamine production in the midbrain (Peterson and Flood, 2012; Hirsch et al., 2013). It is caused by the augmented mitochondrial dysfunction and oxidative stress that might occur due to the selective inhibition of complex I action (Hu et al., 2010; Tapias et al., 2014). Also heredity and environmental factors, calcium overload, immune abnormalities and various neurotoxins could be responsible for the loss of neurons (Abou-Sleiman et al., 2006).

### Resveratrol in Parkinson’s Disease

**FIGURE 4:**
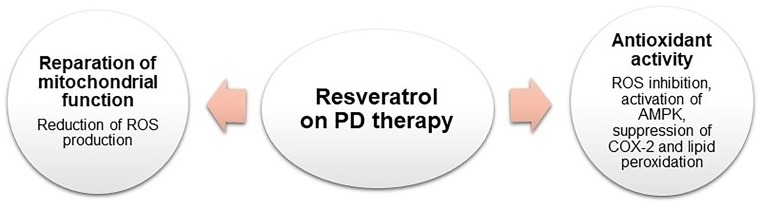
Schematic representation of RES pharmacological activities on PD therapy.

Resveratrol has shown relevant therapeutic effects for the treatment of PD. Numerous findings have proved the RES neuroprotective effects both *in vitro* and on *in vivo* models as presented in Figure 4 (Olanow and Tatton, 1999; Olanow et al., 2004; Sonmez et al., 2007; Jin et al., 2008; Kairisalo et al., 2011; Hauser and Hastings, 2013; Zeng et al., 2017).

#### The Role of Resveratrol in the Oxidative Stress

The oxidative stress has been related with the development of PD. The death of dopaminergic neurons within the *substantia nigra* is observed in PD patients (Olanow and Tatton, 1999; Hauser and Hastings, 2013). It is believed that the death of dopaminergic neurons results from the increase of oxidative stress (Jenner, 2003; Hald and Lotharius, 2005; Henchcliffe and Beal, 2008).

The use of therapeutic molecules to decrease the amount of apoptotic dopaminergic neurons and reduce the oxidative stress in the *substantia nigra* could be a significant strategy for the PD prevention. Reduction of the oxidative stress with specific antioxidants has been proved to be effective (Olanow et al., 2004; Lu et al., 2013). The RES attenuates oxidative stress in PD animal models, demonstrating to be a neuroprotective agent, both *in vitro* and *in vivo* (Jin et al., 2008; Kairisalo et al., 2011; Zeng et al., 2017). Similar to the case of AD, RES acts mainly by inhibiting ROS produced by hippocampal cells to reduce cell death, by activating AMPK (Bastianetto et al., 2000; Virgili and Contestabile, 2000; Nicolini et al., 2001; Lee et al., 2007). Also, it was discovered that RES is able to suppress COX-2 and lipid peroxidation (Szabo, 2009). This molecule increases the expression of some antioxidant enzymes in the brain of healthful animals and presents an excellent antioxidant effect (Mokni et al., 2007).

#### Mitochondrial Dysfunction

Mitochondrial complex I is appointed as one of the main causes of ROS production (Jenner, 2003; Henchcliffe and Beal, 2008). Pathological and biochemical studies in PD animal models presented diseased-specific decrease in protein level or in the substantia nigra pars compacta in mitochondrial complex I activity. Post-mortem brain studies genetic analysis in humans showed similar results (Alam et al., 1997; Floor and Wetzel, 1998). Abnormalities of mitochondrial energy metabolism can induce modification in oxidative homeostasis, increasing the development of free radicals (Saravanan et al., 2005).

Diminution of mitochondrial dysfunction with specific antioxidants, such as RES, has been proved to be effective in *vivo* (Olanow et al., 2004; Jin et al., 2008; Kairisalo et al., 2011; Zeng et al., 2017).

### Nanocarriers for Resveratrol Delivery in Parkinson’s Disease

Despite the efforts to develop a new therapy for PD, the available therapies only attenuate the symptoms. However, they present severe adverse effects (Wang et al., 2016). This type of treatments precludes their long-term use. The treatments with RES strongly suggest that animal models with PD have their symptoms attenuated. Nowadays, a very few approaches have been tested with RES encapsulated into NPs for the treatment of PD (Table 2).

**Table 2:** Currently developed DDS for RES delivery for PD’s therapy.

Carrier	Material	Coating	Targeting ligand	Development phase	Major findings	Reference
Liposomes	Lecithin and cholesterol	No	No	*In vitro*	Enhancement of antioxidant capability of nigral tissues.	Wang et al., 2011
					Decrease of abnormal rotational performance.	
					Apoptosis and loss of nigral cells.	
Polymeric NPs	PLA	Tween 80	No	*In vivo*	Significant neuroprotective effects against MPTP-induced behavioral and neurochemical modifications.	da Rocha Lindner et al., 2015
Nanoemulsion	Vitamin E and sefsol	Tween 80 and Transcutol P	No	*In vitro and ex vivo*	Decrease of degenerative change.	Pangeni et al., 2014
					Increase of the glutathione and superoxide dismutase levels and decrease of the malondialdehyde level.	
NPs	Resveratrol	Hydroxypropyl methylcellulose	No	*In vivo*	Increase of the RES bioavailability and pharmacological activity.	Palle and Neerati, 2018
					Reduction of the behavioral modifications, oxidative stress, mitochondrial dysfunction, and biochemical and histological changes in rats.	

Wang et al. (2011) explored a different approach to protect RES from *Polygonum cuspidatum* in cells of PD rats. RES was encapsulated into liposomes and then tested in nigral cells. This *in vitro* experiment was established by unilateral microinjection of 6-hydroxy dopamine in the striatum. In order to evaluate the RES effect, the formulations containing free RES and RES encapsulated in the liposomes were administered intragastrically. Oral treatment with RES or RES encapsulated in liposomes proved to be beneficial. In fact, the rotational behavior was considerably enhanced. Also, the amount of total ROS, the nigral cells loss and apoptosis decreased. Still, RES encapsulated in liposomes exhibited more intense effects than free RES.

da Rocha Lindner et al. (2015) have explored the neuroprotective effects of RES-loaded polysorbate 80 (PS80)-coated poly(lactide) (PLA) NPs *in vivo* in C57BL/6 mice. During the treatment, the animals received free or encapsulated RES intraperitoneally as well as intranasal administration of 1-methyl-4-phenyl-1,2,3,6-tetrahydropyridine (MPTP). MPTP is a well-known neurotoxin that stimulates PD symptoms and injuries dopaminergic neurons. The MPTP is able to produce disturbances on olfactory taste and recognition memory, in addition to causing striatal oxidative stress and reducing the expression of tyrosine hydroxylase in striatum. In this study the authors observed significant neuroprotective effects beside MPTP-induced behavioral and neurochemical alterations when the animals were treated with RES encapsulated into the NPs. On the other hand, the beneficial effects were not observed in the animals treated with free RES. Herein, PLA NPs coated with PS80 are a promising nanocarrier to transport the RES and consequently treat or prevent PD.

Pangeni et al. (2014) strategy was to formulate a RES loaded Vitamin E nanoemulsion for PD patients brain targeting. The group formulated a kinetically stable nanoemulsion (o/w). To produce this nanoemulsion the authors used vitamin E and propylene glycol mono caprylic ester (Sefsol^®^) with a 1:1 ratio as the oil phase, Tween 80 as the surfactant and Transcutol P as the co-surfactant. Pharmacokinetic studies presented a significant amount of the drug in the brain after intranasal administration of the nanoemulsion. Since the nasal area is very permeable and highly vascularized, the nasal route could be a valuable strategy to administer drugs intended for brain delivery (Pardeshi et al., 2013a,b). Also, the nasal area presents low enzyme levels, great surface area, 150 cm^2^, and high total blood flow per cm^3^. It results in a rapid absorption that could be very beneficial in drug administration. Furthermore, this route is non-invasive and it is an alternative route that effectively bypasses the BBB, circumventing significant intestinal and hepatic metabolism (Haque et al., 2012; Mittal et al., 2014). Results showing a higher RES amount in the brain. This fact confirms the targeting efficiency of the developed nanoemulsion. Histopathological analysis demonstrated a decreased degenerative change in the animal groups administrated with the RES nanoemulsion. The levels of glutathione and superoxide dismutase increased, and the level of malondialdehyde decreased in the group treated with the nanoemulsion.

Recently, Palle and Neerati (2018) studied the effect of RES encapsulated into NPs against rotenone-induced Parkinson’s rats. Some works demonstrated that exposure to pesticides and environmental toxins can lead to an oxidative damage and mitochondrial dysfunction (Swarnkar et al., 2011). Rotenone is a natural organic pesticide, obtained from the plant roots of Leguminosae family. This molecule is able to cross the BBB and causes neurotoxicity by mitochondrial complex-I inhibition (Alam and Schmidt, 2002; Bueler, 2009). In rodents, more specifically in rats, rotenone causes behavioral, biochemical and neuropathological symptoms comparable to the humans’ patients with PD (Betarbet et al., 2000). In this work the authors compared the effect of free and the encapsulated RES. The NPs were produced by temperature-controlled antisolvent precipitation. As stabilizer the hydroxypropyl methylcellulose was used. Here, the rats were separated into four groups: (I) control animals, (II) animals administered with rotenone, (III) animals administered with rotenone and free RES, and (IV) animals administered with rotenone and RES encapsulated in NPs. It was observed that RES encapsulated in NPs could preserve RES in blood for a longer time. That way, with the RES encapsulation it is possible to increase its bioavailability and its pharmacological activity. Behavioral analysis was assessed by rota rod test and rearing behavior. It was observed that chronic rotenone treatment instigated oxidative stress, motor deficits, mitochondrial dysfunction, and decreased rearing behavior. RES encapsulated into NPs presented better efficacy than free RES. In the group treated with encapsulated RES it was observed reduction of the behavioral modifications, oxidative stress, mitochondrial dysfunction, and biochemical and histological changes.

## Glioma Tumors

Gliomas are brain tumors originated from glia cells and they are the type of cancer with the second-highest mortality rate. The type of glioma tumor with the higher incidence, aggressiveness and mortality is the glioblastoma multiforme tumor, that accounts for 77% of brain tumors. The first-line treatment of glioma tumors is based on a multimodal approach combining tumor resection with surgery, chemotherapy and radiotherapy. However, its high proliferation rate and invasiveness contributes to its high resistance to therapy (Bush et al., 2017; Ramalho et al., 2018a). Inhibition of carcinogenesis by RES in glioma tumors can occur at several stages, as inhibition of angiogenesis, induction of apoptosis, tumor growth inhibition, and cell cycle regulation.

### Resveratrol Anticancer Activity in Brain Tumors

**FIGURE 5:**
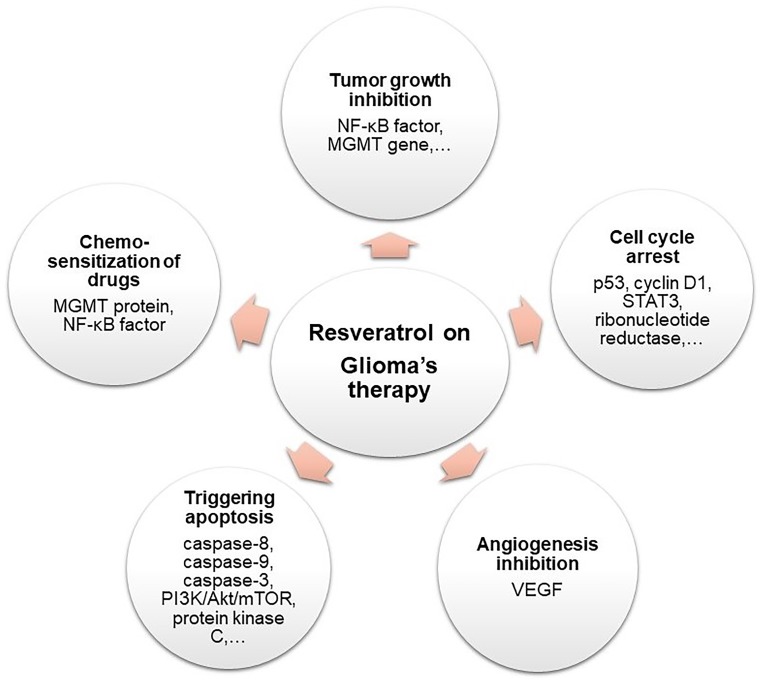
Schematic representation of RES pharmacological activities on glioma therapy.

Resveratrol chemopreventive activity in leukemia cells was reported for the first time in Jang et al. (1997). Since then, RES displayed several anticancer properties in many cellular and animal models for glioma tumors and several mechanisms in which the molecule exerts these anticancer effects have been reported. These mechanisms are presented and resumed below in Figure 5.

#### Carcinogenesis and Cell Cycle Regulation

Several molecules are involved in the carcinogenesis and progression of brain tumors and can be envisaged as therapeutic targets for RES. Tumor suppressor cytoplasmic protein p53 could be one of the crucial targets of RES antiproliferative activity, since most of the glioma tumors have either mutation in p53 or defects in its pathway (Rivlin et al., 2011). P53 inhibits DNA replication in response to stress and DNA damage in mutant cells, by promoting cell cycle arrest at G1/S phase. RES upregulates p53 protein expression by promoting its accumulation and phosphorylation (Clark et al., 2017).

RES also suppresses the progression of the cell cycle by downregulating the expression of enzyme cyclin D1, involved in the transition from the G1 to the S phase of the cell cycle (Pirtoli et al., 2016; Varoni et al., 2016). RES also inhibits the ribonucleotide reductase required for the DNA synthesis at the S-phase of the cell cycle, resulting in the cell cycle arrest (Neves et al., 2012).

Another largely studied target of RES is the transcription factor STAT3. The malignancy and recurrence of glioma tumors is affected by the signaling pathway of STAT3, that is extensively activated by phosphorylation in brain tumors (Attarha et al., 2017). Its role in the carcinogenesis of these tumors includes the regulation of cell cycle progression, mediating the transcription of many different genes, being involved in many cell mechanisms such apoptosis, angiogenesis and tumor invasion of adjacent tissues, acting as an important indicator for the patient’s survival rate, tumor growth, and progression (Yi-Ping et al., 2012).

#### Inhibition of Angiogenesis and Tumor Growth

The invasiveness and malignancy degree of glioma tumors is related with an aberrant neovascularization and the cellular invasion of adjacent tissues. The vascular cell proliferation and invasion to surrounding tissues are regulated by several cytokines and growth factors and depend on a series of molecular events. The vascular endothelial growth factor (VEGF) promotes capillary formation by stimulating endothelial cells proliferation, migration, and invasion into adjacent tissues to form vessels (Smith et al., 2015). Recent studies showed that RES inhibits VEGF expression and activity in glioma cells, suppressing angiogenesis (Tseng et al., 2004; Wang et al., 2015).

The proteolysis of the extracellular matrix is also a crucial event for the infiltrative growth of glioma. RES was found to suppress NF-κB factor activation by hampering the nuclear translocation of its p65 subunit, leading to a downregulation on the expression of uPA/uPAR genes reducing the glioma cell invasion (Ryu et al., 2011).

#### Promoting Apoptosis

Programmed cell death – apoptosis – causes cell changes, as cell shrinkage, loss of cell membrane integrity, and DNA fragmentation causing the cellular death. Apoptosis can be induced by several intra and extracellular factors and the absence of apoptotic responses is crucial to the development of tumors and resistance to therapy (Brown and Attardi, 2005). RES is reported to induce apoptosis by several mechanisms in glioma cells. In fact, Tseng et al. (2004) showed that RES induced apoptosis of glioma cells on rat models. RES can induce apoptosis either through the caspase-8-dependent or the caspase-9-dependent pathway in U251, U87, and C6 glioma cells (Jiang et al., 2005; Zhang et al., 2007; Filippi-Chiela et al., 2011). Both are initiator caspases responsible for the activation of effector caspase-3 (by hydrolytic cleavage), that will disassemble glioma cells into apoptotic bodies (Aggarwal et al., 2004). RES also promotes apoptosis by downregulating the expression of survivin, an inhibitor of the effector caspases (Hayashibara et al., 2002). Recent studies show that RES can promote caspase-3 activation by inhibiting the PI3K/Akt/mTOR intracellular signaling pathway in glioma cells (Jiang et al., 2009a,b). The PI3K/Akt/mTOR pathway is overactive in glioma tumors, resulting in reduced apoptosis and increased tumor proliferation, being frequently implicated in resistance to anticancer therapy (Burris, 2013).

RES can also induce apoptosis by suppressing another molecular mechanism, the signaling pathway of protein kinase C (Couldwell et al., 1991). It is reported that protein kinase C is overexpressed in glioma cells and is related with cellular proliferation (Tseng et al., 2004).

#### Chemosensitization of Other Anticancer Drugs as Temozolomide

Temozolomide is the standard drug used for glioblastoma therapy, however, its use is highly ineffective due to several resistance mechanisms and biological barriers (Mallick et al., 2015). *O*(6)-Methylguanine DNA-methyltransferase (MGMT) protein is related to resistance to therapy with temozolomide, since it repairs the DNA damage caused by temozolomide-induced methylation. Huang and colleagues showed that RES decreased the expression of the MGMT protein in glioblastoma cells, by suppressing the activation of the NF-κB transcription factor essential for MGMT activation. Thus, RES reverses the resistance to therapy and increases the efficiency of temozolomide (Huang et al., 2012).

Several other authors showed that RES enhanced chemosensitization of glioma cells to temozolomide activity, by altering multiple signaling pathways and promoting apoptosis (Lin et al., 2012; Yuan et al., 2012; Li et al., 2016) and cell cycle arrest (Filippi-Chiela et al., 2013).

### Nanocarriers for Resveratrol Delivery in Glioma Treatment

All the find data, showing a slower tumor growth rate and prolonged animal survival time, suggest that prolonged treatment with RES is a suitable strategy for the therapy of glioma tumors. However due to the limitations associated to its use, nanobased strategies could be envisaged to increase its bioavailability and activity. Several nanotechnology approaches have been proposed in the last decade for glioma tumors’ therapy with several different drugs and nanomaterials (Li et al., 2014; Householder et al., 2015; Yao et al., 2015; Ramalho et al., 2018b). In this section we focused on the DDS already reported for the encapsulation and brain delivery of RES to glioma tumor cells (Table 3).

**Table 3:** Currently developed DDS for RES delivery for glioma’s therapy.

Carrier	Material	Coating	Targeting ligand	Development phase		Reference
Polymeric NPs	PLGA	TPGS	No	*In vivo*	Longer Increased system circulation time and increased brain accumulation.	Vijayakumar et al., 2016a
		No	Folic acid	*In vivo*	Improved intracellular biodistribution of RES. Retained RES ability of tumor growth inhibition.	Xin et al., 2016
	PLA	PEG	Transferrin	*In vivo*	Increased cellular uptake, and accumulation in the brain of glioma tumor xenograft-bearing rats.	Guo et al., 2013
	PCL	PEG	No	*In vitro*	Synergistically enhanced the cytotoxicity of an alkylating agent.	Shao et al., 2009; Xu et al., 2017
Polymeric nanofibers	PCL	mPEG	No	*In vivo*	Enhanced antiproliferative activity.	Zhou et al., 2016
Lipid-core NCs	Capric/caprylic triglyceride and sorbitan monostearate	No	No	*In vivo*	Improved RES ability to suppress tumor growth.	Figueiró et al., 2013
Liposomes	Phosphatidylcholine, DSPE and cholesterol	PEG	No	*In vivo*	Longer systemic circulation time and higher accumulation in brain tissue.	Vijayakumar et al., 2016b
		PEG and TPGS	No	*In vivo*	Increased accumulation in brain tissue and increased NPs’ biocompatibility.	Vijayakumar et al., 2016d
	DOPE	PEG	Transferrin	*In vivo*	Enhanced cellular uptake and improved tumor growth inhibition in glioblastoma tumor heterotopic xenograft-bearing mice.	Jhaveri et al., 2018
SLNs	Compritol 888 ATO^®^	PVA or Tween 80	No	*In vivo*	Increased accumulation of RES in rats’ brains.	Jose et al., 2014
	Tristearin and soyaphosphotidyl choline	TPGS	No	*In vivo*	Longer Increased system circulation time and increased brain accumulation.	Vijayakumar et al., 2016c

Shao and colleagues proposed for the first time the entrapment of RES for glioma therapy. The group developed NPs composed of methoxy poly(ethylene glycol)-poly(caprolactone), (mPEG-PCL). The NPs were prepared by nanoprecipitation. The nanosystems had approximately 80 nm, low and negative zeta potential and high loading efficiency values (about 90%). The NPs were evaluated i*n vitro*, using rat glioma cells. The attained results showed that the NPs are efficiently internalized by the target cells and indicated that nanoencapsulation of RES did not decreased its antiproliferative activity. The developed nanosystem exhibits similar toxicity to free RES at high concentrations, but superior toxicity at lower doses, suggesting that this could be a suitable strategy for glioma treatment, since encapsulated RES retains its ability to increase intracellular ROS levels, inhibiting the growth of glioma tumors (Shao et al., 2009). A few years later, the group proposed the use of this developed nanosystem for the co-delivery of RES with temozolomide, to increase the cellular chemosensitivity to the alkylating agent. The nanosystems exhibited increased mean dimensions due to the entrapment of both drugs (130 nm), still low negative zeta potential and encapsulation efficiency values of about 80% for both drugs. *In vitro* studies were conducted with human glioblastoma cell lines and showed an efficient uptake and that RES synergistically enhanced the cytotoxicity of temozolomide. Encapsulation of RES in the developed nanocarrier enhanced apoptotic induction and inhibition of migration and invasion. The co-delivery of both drugs also resulted in a more efficient tumor growth inhibition than the combination of both free drugs, or each drug alone. The attained results proved that the dual-delivery of these drugs could be a suitable strategy for glioblastoma therapy (Xu et al., 2017).

Figueiró and colleagues evaluated the efficiency of the previously developed NCs (Frozza et al., 2010) in rat glioma cells and in glioma tumor orthotopic xenograft-bearing rats. The authors verified that encapsulated RES decreased *in vitro* cellular viability more efficiently than its free form. The group also concluded that encapsulated RES maintains the ability of induction of apoptosis and cell cycle arrest. Animal studies showed that encapsulation of RES allowed a decrease in the dimensions of the tumor as well as the reduction in the occurrence of some tumor malignancy characteristics, indicating a less aggressive, invasive and proliferative tumor (Figueiró et al., 2013).

Guo and colleagues developed pegylated PLA NPs using a single water-in-oil emulsion technique. The attained PLA NPs had about 150 nm, net negative charge and high encapsulation efficiency of around 80%. Transferrin was added to the surface of the NPs. Since its receptor is overexpressed in glioma tumor cells and in the endothelial cells of the BBB, the nanosystem envisages a dual-targeting strategy for tumor brain delivery. *In vitro* studies showed that encapsulated RES had much higher cytotoxicity as compared with its free form, in both rat glioma and human glioblastoma cell lines, since it enhanced the activation of an effector caspase, increasing apoptosis rate. Modification with transferrin proved to be a suitable strategy since it increased cellular uptake in the studied cell lines, and drug accumulation in the brain of glioma tumor xenograft-bearing rats (Guo et al., 2013).

Jose and co-workers prepared compritol 888 ATO^®^ SLN using the solvent evaporation, high speed homogenization and ultrasonic techniques (Jose et al., 2014). The group evaluated the influence of drug-lipid ratio and used stabilizer [tween 80 or polyvinyl alcohol (PVA)] on physicochemical properties of the NPs and their encapsulation efficiency. The most adequate nanoformulation had sizes of about 250 nm, negative charge and low entrapment efficiency, being able to encapsulate only about 35% of the used RES. *In vitro* studies using rat glioma cells showed a similar cytotoxicity in encapsulated RES and in the free form of the drug. However, the developed nanoformulation is advantageous since it presented an higher drug accumulation in the rats brain, proving that this nanosystem is able to bypass the BBB (Jose et al., 2014).

Vijakumar and colleagues proposed the use of liposomes for the first time for glioma tumor therapy with RES. The liposomes composed of phosphatidylcholine, DSPE-PEG and cholesterol were prepared by the by thin-film hydration methodology. The nanoformulation presented mean dimensions of 250 nm, negative superficial charge and about 80% of loading efficiency. The developed liposomes showed longer systemic circulation time and higher accumulation in brain tissue than free RES, proving to that this nanoformulation is an efficient nanocarrier for the brain delivery (Vijayakumar et al., 2016b). Later in the same year, Vijayakumar and co-workers presented an improved liposomal nanoformulation for the delivery of RES. The liposomes were coated with D-α-tocopherol polyethylene glycol 1000 succinate (TPGS) to increase the bloodstream circulation time and brain delivery of RES. Several drug/lipid ratios and sonication time were evaluated, and the most suitable nanoformulation was chosen. The optimal formulation exhibited small dimensions (65 nm), neutral charge and an entrapment efficiency of almost 80%. The RES nanoformulations were evaluated *in vitro* and *in vivo*. Experiments using rat glioma cells showed significantly higher cytotoxicity for encapsulated RES. Additionally, biodistribution tests in rats showed an increase in the amount of RES in the brain when delivered by the liposomes, indicating the passive brain targeting potential of the nanoformulation. The coating of liposomes with TPGS prolonged the systemic circulation of RES, showing no platelet aggregation or hemolysis (Vijayakumar et al., 2016d).

The authors again employed the same strategy to increase systemic circulation and passive brain targeting of RES with different types of NPs for glioma therapy. They proposed the use of poly(lactic-co-glycolic acid) (PLGA) and SLN. Tristearin and soyaphosphotidyl choline lipids were used for the nanoformulation of SLN prepared by the solvent emulsification evaporation method. Several experimental parameters were evaluated, and the most adequate nanoformulation exhibited sizes of about 200 nm, negative surface charge and entrapment efficiency values of about 70% (Vijayakumar et al., 2016c). The PLGA NPs developed by Vijayakumar and co-workers were prepared using the single-emulsion method. The influence of different ratios of organic to aqueous solvent and polymer/TPGS on these NPs physicochemical properties were also assessed. The optimal nanoformulation was chosen (size of 175 nm, negative superficial charge and loading efficiency of about 60%) (Vijayakumar et al., 2016a). Encapsulated RES in both PLGA NPs and SLN showed higher cytotoxicity than its free form in rat glioma cells. When RES molecules were delivered by the nanocarriers, a higher accumulation of the drug in the brain of the rats was verified. These nanoformulations also proved to increase system circulation time and enhance passive brain targeting, while being hemocompatible and safe after intravenous administration (Vijayakumar et al., 2016a,c).

Zhou and co-workers developed mPEG-PCL block copolymer nanofibers for RES delivery in glioblastoma therapy. *In vitro* experiments with a glioblastoma cellular line obtained from human patients showed an efficient cellular internalization of the nanofibers and higher cytotoxicity for encapsulated RES for long incubation periods. For short incubation periods, free RES exhibited higher toxicity to cells, due to the slow release of RES from nanofibers. Encapsulation of RES in nanofibers retained drug ability to promote apoptosis and delay cell migration and invasion. Finally, nanodelivery of RES enhanced its efficiency in decreasing the tumor in mice (Zhou et al., 2016).

Jhaveri et al. (2018) developed negatively charged liposomes using the thin film hydration method. The prepared liposomes were smaller than 200 nm and presented a high encapsulation of the drug of around 70%. The nanosystems surface was modified using transferrin molecules to enhance uptake from glioblastoma cells. Although, authors did not mention the intent to target BBB, since transferrin receptor is also overexpressed in endothelial cells of this barrier, the system could foresee a dual-targeting strategy. Also, the developed liposomes exhibited suitable physicochemical properties (size and charge) to allow their passage across the BBB. *In vitro* competitive binding experiments with glioblastoma cells from humans proved that modification with transferrin molecules enhanced the cellular uptake by taking advantage of the transferrin receptor-mediated endocytic pathway. This increased internalization justifies the observed higher efficiency in decreasing cellular viability of RES when encapsulated in transferrin-modified liposomes, comparatively with non-modified nanosystems. The authors showed that encapsulated RES retained its ability to halt the progression of the cell cycle and to induce apoptotic cell death by activation of effector caspases. The prepared RES-liposomes improved tumor growth inhibition and survival in glioblastoma tumor heterotopic xenograft-bearing mice, proving that the proposed systems is suitable for therapy (Jhaveri et al., 2018).

Xin colleagues proposed for the first time the encapsulation of RES in a nanosystem envisaged for glioma therapy and diagnostic simultaneous application. PLGA NPs were prepared using the nanoprecipitation method. The developed NPs were conjugated with indocyanine green (ICG), a clinical FDA-approved fluorescence imaging probe for tumor diagnosis, and folic acid for tumor cell targeting. DSPE-PEG was used for ICG conjugation. The obtained PLGA NPs with 120 nm and negative surface charge showed a high entrapment efficiency (almost 70%). *In vitro* experiments, on glioblastoma cells obtained from human patients, proved that the NPs are efficiently uptaken through folic acid receptor-mediated endocytic mechanism. Cytotoxicity studies also showed higher antiproliferative activity and higher apoptosis rate when RES was encapsulated. *In vivo* studies with heterotopic xenograft tumor-bearing mice proved that the nanoformulation is able to delay the elimination of ICG and RES, increasing the systemic circulation time. Biodistribution studies showed that encapsulation resulted in a higher accumulation of RES and ICG in brain tissue. The authors also concluded that encapsulation enhanced tumor growth inhibition and fluorescent signal of ICG, proving that the proposed nanocarrier could be a suitable *in vivo* theranostics tool (Xin et al., 2016).

## Strategies to Improve the Efficiency of the DDS to Deliver RES Into the Brain

Several approaches have been studied in the last years to delivery RES to the brain. Different types of materials have been used to encapsulate RES and improve its activity. Some of the reported DDS present advantageous characteristics, such as enhanced stability, bioavailability, and biocompatibility. It is well-known that after administration, the NPs could be recognized by the immune systems being removed from the systemic circulation and being eliminated in the liver or spleen (Schroeder et al., 1998; Wohlfart et al., 2012). Some strategies can be used to increase the bloodstream circulation time of the NPs. One of the most popular strategies is the attachment or adsorption of different molecules to the NPs’ surface. Hydrophilic stabilizers, such as PEG and polysorbates can be used. These molecules also provide steric stabilization to the surface of the NPs and allow the attachment of other ligand moieties such as antibodies, aptamers, and proteins recognized by the receptors at the BBB cells (Masserini, 2013). However, these ligand molecules can facilitate the NPs recognition by the immune system and promote their elimination. Though, the coating the NPs’ surface with PEG molecules allows reducing this phenomenon (Chen and Liu, 2012).

The attachment of targeting molecules to the NP’s surface can also be used as strategy to enhance the bioavailability of the encapsulated RES. The functionalization of the NPs with these ligand moieties allows the brain tissue targeting, since they are recognized by the existent receptors at the BBB, enabling the NP’s active transport across this barrier. Also using positively charged targeting ligands, their interaction with the luminal surface of the cells of the BBB is promoted by electrostatic forces (Pehlivan, 2013). The ligand must be carefully chosen since its receptor must be overexpressed at the BBB, and also should be preferentially specific to the brain tissue to reduce harmful effects to the healthy tissues and increase RES accumulation in the brain (Chen and Liu, 2012). The saturation of the receptor must also be carefully evaluated to circumvent competitive-binding of the natural ligand (Masserini, 2013). Although none of the reported NPs for the delivery of RES in the AD and PD treatment used specific molecular and cellular targets for these diseases, some targeting moieties can be explored such as α-synuclein protein, the parkin protein and leucine-rich repeat serine/threonine protein kinase 2 for PD, and α-, β-, and γ-synuclein peptides for AD (Garbayo et al., 2013). However, brain targeting was achieved by using specific ligands for BBB.

In fact, several of the reported works for RES encapsulation for brain delivery demonstrate increased physicochemical features of the DDS when coating and targeting strategies are incorporated.

## Conclusion and Future Perspectives

Resveratrol displays several pharmacological properties, that include anti-inflammatory, anti-oxidant, and anti-tumor functions. However, its biopharmaceutical and pharmacokinetic issues limit its use as a therapeutic agent. RES is mainly insoluble in water and gastrointestinal fluids and has low oral absorption, resulting in a low bioavailability, limiting its clinical application.

In this context, the use of NPs represents an advantageous approach to transport RES to its site of action. The drug pharmacokinetics is affected by the physicochemical properties of the NPs, conditioning their bioavailability and biodistribution. Also, the majority of the NPs have high stability and loading capacity. Furthermore, the NPs exhibit a controlled release profile.

Various strategies using different nanomaterials were proposed for delivery of RES to prevent and treat different neurological diseases, including AD, PD, and glioma. These nanosystems containing RES proved to be effective *in vitro* and *in vivo*. It is expected to continue to improve these NPs to ultimately test them in preclinical and clinical trials.

In the next years, it is expected to continue developing different types of NPs with surface modifications for RES brain targeting.

## Author Contributions

SA and MR wrote the manuscript. MP and JL also contributed with the paper organization. All the authors contributed with the bibliographic research.

## Conflict of Interest Statement

The authors declare that the research was conducted in the absence of any commercial or financial relationships that could be construed as a potential conflict of interest.

## References

[B1] Abolfathi A. A. Mohajeri D. Rezaie A. Nazeri M. (2012). Protective effects of green tea extract against hepatic tissue injury in streptozotocin-induced diabetic rats. *Evid. Based Complement. Alternat. Med.* 2012:740671. 10.1155/2012/740671 22956978 PMC3432555

[B2] Abou-Sleiman P. M. Muqit M. M. Wood N. W. (2006). Expanding insights of mitochondrial dysfunction in Parkinson’s disease. *Nat. Rev. Neurosci.* 7 207–219. 10.1038/nrn1868 16495942

[B3] Aggarwal B. B. Bhardwaj A. Aggarwal R. S. Seeram N. P. Shishodia S. Takada Y. (2004). Role of resveratrol in prevention and therapy of cancer: preclinical and clinical studies. *Anticancer Res.* 24 2783–2840. 15517885

[B4] Ahmed M. Davis J. Aucoin D. Sato T. Ahuja S. Aimoto S. (2010). Structural conversion of neurotoxic amyloid-beta(1-42) oligomers to fibrils. *Nat. Struct. Mol. Biol.* 17 561–567. 10.1038/nsmb.1799 20383142 PMC2922021

[B5] Ahmed T. Javed S. Javed S. Tariq A. Šamec D. Tejada S. (2017). Resveratrol and Alzheimer’s disease: mechanistic insights. *Mol. Neurobiol.* 54 2622–2635. 10.1007/s12035-016-9839-9 26993301

[B6] Ahn J. S. Lee J. H. Kim J.-H. Paik S. R. (2007). Novel method for quantitative determination of amyloid fibrils of α-synuclein and amyloid β/A4 protein by using resveratrol. *Anal. Biochem.* 367 259–265. 10.1016/j.ab.2007.05.023 17597573

[B7] Alam M. Schmidt W. J. (2002). Rotenone destroys dopaminergic neurons and induces parkinsonian symptoms in rats. *Behav. Brain Res.* 136 317–324. 10.1016/S0166-4328(02)00180-8 12385818

[B8] Alam Z. I. Jenner A. Daniel S. E. Lees A. J. Cairns N. Marsden C. D. (1997). Oxidative DNA damage in the parkinsonian brain: an apparent selective increase in 8-hydroxyguanine levels in *Substantia nigra*. *J. Neurochem.* 69 1196–1203. 10.1046/j.1471-4159.1997.69031196.x 9282943

[B9] Albani D. Polito L. Batelli S. De Mauro S. Fracasso C. Martelli G. (2009). The SIRT1 activator resveratrol protects SK-N-BE cells from oxidative stress and against toxicity caused by alpha-synuclein or amyloid-beta (1-42) peptide. *J. Neurochem.* 110 1445–1456. 10.1111/j.1471-4159.2009.06228.x 19558452

[B10] Al-Bishri W. M. Hamza A. H. Farran S. K. (2017). Resveratrol treatment attenuates amyloid beta, Tau protein and markers of oxidative stress, and inflammation in Alzheimer’s disease rat model. *Int. J. Pharm. Res. Allied Sci.* 6 71–78.

[B11] Amri A. Chaumeil J. C. Sfar S. Charrueau C. (2012). Administration of resveratrol: what formulation solutions to bioavailability limitations? *J. Control. Release* 158 182–193. 10.1016/j.jconrel.2011.09.083 21978644

[B12] Anandatheerthavarada H. K. Biswas G. Robin M. A. Avadhani N. G. (2003). Mitochondrial targeting and a novel transmembrane arrest of Alzheimer’s amyloid precursor protein impairs mitochondrial function in neuronal cells. *J. Cell Biol.* 161 41–54. 10.1083/jcb.200207030 12695498 PMC2172865

[B13] Andrade S. Loureiro J. A. Coelho M. A. N. Pereira M. D. C. (2015). “Interaction studies of amyloid beta-peptide with the natural compound resveratrol,” in *Proceedings of the 4th Portuguese Meeting on Bioengineering (ENBENG)*, (Porto: IEEE), 1–3. 10.1109/ENBENG.2015.7088833

[B14] Anekonda T. S. (2006). Resveratrol—A boon for treating Alzheimer’s disease? *Brain Res. Rev.* 52 316–326. 16766037 10.1016/j.brainresrev.2006.04.004

[B15] Arendt T. (2009). Synaptic degeneration in Alzheimer’s disease. *Acta Neuropathol.* 118 167–179. 10.1007/s00401-009-0536-x 19390859

[B16] Arora D. Jaglan S. (2017). “Nanocarriers for resveratrol delivery,” in *Nanoscience in Food and Agriculture* Vol. 5 eds Ranjan S. Dasgupta N. Lichtfouse E. (Cham: Springer International Publishing), 123–138. 10.1007/978-3-319-58496-6_5

[B17] Arriagada P. V. Growdon J. H. Hedley-Whyte E. T. Hyman B. T. (1992). Neurofibrillary tangles but not senile plaques parallel duration and severity of Alzheimer’s disease. *Neurology* 42 631–639. 10.1212/WNL.42.3.631 1549228

[B18] Attarha S. Roy A. Westermark B. Tchougounova E. (2017). Mast cells modulate proliferation, migration and stemness of glioma cells through downregulation of GSK3β expression and inhibition of STAT3 activation. *Cell. Signal.* 37 81–92. 10.1016/j.cellsig.2017.06.004 28600192

[B19] Ballard C. Gauthier S. Corbett A. Brayne C. Aarsland D. Jones E. (2011). Alzheimer’s disease. *Lancet* 377 1019–1031. 10.1016/S0140-6736(10)61349-9 21371747

[B20] Bastianetto S. Menard C. Quirion R. (2015). Neuroprotective action of resveratrol. *Biochim. Biophys. Acta* 1852 1195–1201. 10.1016/j.bbadis.2014.09.011 25281824

[B21] Bastianetto S. Quirion R. (2010). Heme oxygenase 1: another possible target to explain the neuroprotective action of resveratrol, a multifaceted nutrient-based molecule. *Exp. Neurol.* 225 237–239. 10.1016/j.expneurol.2010.06.019 20603117

[B22] Bastianetto S. Zheng W. H. Quirion R. (2000). Neuroprotective abilities of resveratrol and other red wine constituents against nitric oxide-related toxicity in cultured hippocampal neurons. *Br. J. Pharmacol.* 131 711–720. 10.1038/sj.bjp.0703626 11030720 PMC1572384

[B23] Bates K. A. Verdile G. Li Q. X. Ames D. Hudson P. Masters C. L. (2009). Clearance mechanisms of Alzheimer’s amyloid-beta peptide: implications for therapeutic design and diagnostic tests. *Mol. Psychiatry* 14 469–486. 10.1038/mp.2008.96 18794889

[B24] Berman A. Y. Motechin R. A. Wiesenfeld M. Y. Holz M. K. (2017). The therapeutic potential of resveratrol: a review of clinical trials. *NPJ Precis. Oncol.* 1:35. 10.1038/s41698-017-0038-6 28989978 PMC5630227

[B25] Betarbet R. Sherer T. B. MacKenzie G. Garcia-Osuna M. Panov A. V. Greenamyre J. T. (2000). Chronic systemic pesticide exposure reproduces features of Parkinson’s disease. *Nat. Neurosci.* 3 1301–1306. 10.1038/81834 11100151

[B26] Bhowmik A. Khan R. Ghosh M. K. (2015). Blood brain barrier: a challenge for effectual therapy of brain tumors. *Biomed Res. Int.* 2015:320941. 10.1155/2015/320941 25866775 PMC4383356

[B27] Blanco E. Shen H. Ferrari M. (2015). Principles of nanoparticle design for overcoming biological barriers to drug delivery. *Nat. Biotechnol.* 33 941–951. 10.1038/nbt.3330 26348965 PMC4978509

[B28] Brookmeyer R. Johnson E. Ziegler-Graham K. Arrighi H. M. (2007). Forecasting the global burden of Alzheimer’s disease. *Alzheimers Dement.* 3 186–191. 10.1016/j.jalz.2007.04.381 19595937

[B29] Brown J. M. Attardi L. D. (2005). The role of apoptosis in cancer development and treatment response. *Nat. Rev. Cancer* 5 231–237. 10.1038/nrc1560 15738985

[B30] Bueler H. (2009). Impaired mitochondrial dynamics and function in the pathogenesis of Parkinson’s disease. *Exp. Neurol.* 218 235–246. 10.1016/j.expneurol.2009.03.006 19303005

[B31] Burris H. A. (2013). Overcoming acquired resistance to anticancer therapy: focus on the PI3K/AKT/mTOR pathway. *Cancer Chemother. Pharmacol.* 71 829–842. 10.1007/s00280-012-2043-3 23377372

[B32] Bush N. A. O. Chang S. M. Berger M. S. (2017). Current and future strategies for treatment of glioma. *Neurosurg. Rev.* 40 1–14. 10.1007/s10143-016-0709-8 27085859

[B33] Capiralla H. Vingtdeux V. Zhao H. Sankowski R. Al-Abed Y. Davies P. (2012). Resveratrol mitigates lipopolysaccharide- and Abeta-mediated microglial inflammation by inhibiting the TLR4/NF-kappaB/STAT signaling cascade. *J. Neurochem.* 120 461–472. 10.1111/j.1471-4159.2011.07594.x 22118570 PMC3253186

[B34] Cardoso F. L. Brites D. Brito M. A. (2010). Looking at the blood–brain barrier: molecular anatomy and possible investigation approaches. *Brain Res. Rev.* 64 328–363. 10.1016/j.brainresrev.2010.05.003 20685221

[B35] Carneiro P. Loureiro J. Delerue-Matos C. Morais S. do Carmo Pereira M. (2017). Alzheimer’s disease: development of a sensitive label-free electrochemical immunosensor for detection of amyloid beta peptide. *Sens. Actuators B Chem.* 239 157–165. 10.1016/j.snb.2016.07.181

[B36] Chen M. L. Yi L. Jin X. Liang X. Y. Zhou Y. Zhang G. (2013). Resveratrol attenuates vascular endothelial inflammation by inducing autophagy through the cAMP signaling pathway. *Autophagy* 9 2033–2045. 10.4161/auto.26336 24145604

[B37] Chen Y. Liu L. (2012). Modern methods for delivery of drugs across the blood–brain barrier. *Adv. Drug Deliv. Rev.* 64 640–665. 10.1016/j.addr.2011.11.010 22154620

[B38] Chopra P. Hao J. Li S. K. (2013). Influence of drug lipophilicity on drug release from sclera after iontophoretic delivery of mixed micellar carrier system to human sclera. *J. Pharm. Sci.* 102 480–488. 10.1002/jps.23370 23150488 PMC3815607

[B39] Clark P. A. Bhattacharya S. Elmayan A. Darjatmoko S. R. Thuro B. A. Yan M. B. (2017). Resveratrol targeting of AKT and p53 in glioblastoma and glioblastoma stem-like cells to suppress growth and infiltration. *J. Neurosurg.* 126 1448–1460. 10.3171/2016.1.JNS152077 27419830 PMC5237623

[B40] Clippingdale A. B. Wade J. D. Barrow C. J. (2001). The amyloid-beta peptide and its role in Alzheimer’s disease. *J. Pept. Sci.* 7 227–249. 10.1002/psc.324 11428545

[B41] Couldwell W. T. Uhm J. H. Antel J. P. Yong V. W. (1991). Enhanced protein kinase C activity correlates with the growth rate of malignant gliomas in vitro. *Neurosurgery* 29 880–887. 10.1227/00006123-199112000-00013 1758601

[B42] da Rocha Lindner G. Bonfanti Santos D. Colle D. Gasnhar Moreira E. L. Daniel Prediger R. Farina M. (2015). Improved neuroprotective effects of resveratrol-loaded polysorbate 80-coated poly(lactide) nanoparticles in MPTP-induced Parkinsonism. *Nanomedicine* 10 1127–1138. 10.2217/nnm.14.165 25929569

[B43] de Almeida L. M. Pineiro C. C. Leite M. C. Brolese G. Tramontina F. Feoli A. M. (2007). Resveratrol increases glutamate uptake, glutathione content, and S100B secretion in cortical astrocyte cultures. *Cell. Mol. Neurobiol.* 27 661–668. 10.1007/s10571-007-9152-2 17554623 PMC11517203

[B44] Deane R. Wu Z. Sagare A. Davis J. Du Yan S. Hamm K. (2004). LRP/amyloid beta-peptide interaction mediates differential brain efflux of Abeta isoforms. *Neuron* 43 333–344. 10.1016/j.neuron.2004.07.017 15294142

[B45] Donmez G. Wang D. Cohen D. E. Guarente L. (2010). SIRT1 suppresses beta-amyloid production by activating the alpha-secretase gene ADAM10. *Cell* 142 320–332. 10.1016/j.cell.2010.06.020 20655472 PMC2911635

[B46] Draczynska-Lusiak B. Chen Y. M. Sun A. Y. (1998). Oxidized lipoproteins activate NF-kappaB binding activity and apoptosis in PC12 cells. *Neuroreport* 9 527–532. 10.1097/00001756-199802160-00028 9512401

[B47] El-Sayed N. S. Bayan Y. (2015). Possible role of resveratrol targeting estradiol and neprilysin pathways in lipopolysaccharide model of Alzheimer disease. *Adv. Exp. Med. Biol.* 822 107–118. 10.1007/978-3-319-08927-0_12 25416980

[B48] Ethemoglu M. S. Seker F. B. Akkaya H. Kilic E. Aslan I. Erdogan C. S. (2017). Anticonvulsant activity of resveratrol-loaded liposomes in vivo. *Neuroscience* 357 12–19. 10.1016/j.neuroscience.2017.05.026 28577913

[B49] Feng X. Liang N. Zhu D. Gao Q. Peng L. Dong H. (2013). Resveratrol inhibits beta-amyloid-induced neuronal apoptosis through regulation of SIRT1-ROCK1 signaling pathway. *PLoS One* 8:e59888. 10.1371/journal.pone.0059888 23555824 PMC3610881

[B50] Feng Y. Wang X. P. Yang S. G. Wang Y. J. Zhang X. Du X. T. (2009). Resveratrol inhibits beta-amyloid oligomeric cytotoxicity but does not prevent oligomer formation. *Neurotoxicology* 30 986–995. 10.1016/j.neuro.2009.08.013 19744518

[B51] Figueiró F. Bernardi A. Frozza R. L. Terroso T. Zanotto-Filho A. Jandrey E. H. F. (2013). Resveratrol-loaded lipid-core nanocapsules treatment reduces in vitro and in vivo glioma growth. *J. Biomed. Nanotechnol.* 9 516–526. 10.1166/jbn.2013.1547 23621009

[B52] Filippi-Chiela E. C. Thom M. P. Bueno e Silva M. M. Pelegrini A. L. Ledur P. F. Garicochea B. (2013). Resveratrol abrogates the Temozolomide-induced G2 arrest leading to mitotic catastrophe and reinforces the Temozolomide-induced senescence in glioma cells. *BMC Cancer* 13:147. 10.1186/1471-2407-13-147 23522185 PMC3635906

[B53] Filippi-Chiela E. C. Villodre E. S. Zamin L. L. Lenz G. (2011). Autophagy interplay with apoptosis and cell cycle regulation in the growth inhibiting effect of resveratrol in glioma cells. *PLoS One* 6:e20849. 10.1371/journal.pone.0020849 21695150 PMC3113895

[B54] Floor E. Wetzel M. G. (1998). Increased protein oxidation in human *Substantia nigra* pars compacta in comparison with basal ganglia and prefrontal cortex measured with an improved dinitrophenylhydrazine assay. *J. Neurochem.* 70 268–275. 10.1046/j.1471-4159.1998.70010268.x 9422371

[B55] Francioso A. Mastromarino P. Masci A. Erme M. Mosca L. (2014). Chemistry, stability and bioavailability of resveratrol. *Med. Chem.* 10 237–245. 10.2174/15734064113096660053 24329932

[B56] Frozza R. L. Bernardi A. Hoppe J. B. Meneghetti A. B. Battastini A. M. O. Pohlmann A. R. (2013a). Lipid-core nanocapsules improve the effects of resveratrol against Abeta-induced neuroinflammation. *J. Biomed. Nanotechnol.* 9 2086–2104. 10.1166/jbn.2013.1709 24266263

[B57] Frozza R. L. Bernardi A. Hoppe J. B. Meneghetti A. B. Matté A. Battastini A. M. O. (2013b). Neuroprotective effects of resveratrol against Aβ administration in rats are improved by lipid-core nanocapsules. *Mol. Neurobiol.* 47 1066–1080. 10.1007/s12035-013-8401-2 23315270

[B58] Frozza R. L. Bernardi A. Paese K. Hoppe J. B. Silva T. D. Battastini A. M. O. (2010). Characterization of trans-resveratrol-loaded lipid-core nanocapsules and tissue distribution studies in rats. *J. Biomed. Nanotechnol.* 6 694–703. 10.1166/jbn.2010.1161 21361135

[B59] Fu Z. Aucoin D. Ahmed M. Ziliox M. Van Nostrand W. E. Smith S. O. (2014). Capping of abeta42 oligomers by small molecule inhibitors. *Biochemistry* 53 7893–7903. 10.1021/bi500910b 25422864 PMC4278677

[B60] Garbayo E. Ansorena E. Blanco-Prieto M. J. (2013). Drug development in Parkinson’s disease: from emerging molecules to innovative drug delivery systems. *Maturitas* 76 272–278. 10.1016/j.maturitas.2013.05.019 23827471

[B61] Ge J. F. Qiao J. P. Qi C. C. Wang C. W. Zhou J. N. (2012). The binding of resveratrol to monomer and fibril amyloid beta. *Neurochem. Int.* 61 1192–1201. 10.1016/j.neuint.2012.08.012 22981725

[B62] Ghersi-Egea J. F. Mönkkönen K. S. Schmitt C. Honnorat J. Fèvre-Montange M. Strazielle N. (2009). Blood–brain interfaces and cerebral drug bioavailability. *Rev. Neurol.* 165 1029–1038. 10.1016/j.neurol.2009.09.011 19913860

[B63] Goedert M. Klug A. Crowther R. A. (2006). Tau protein, the paired helical filament and Alzheimer’s disease. *J. of Alzheimers Dis.* 9 195–207. 10.3233/JAD-2006-9S323 16914859

[B64] Gomes B. Loureiro J. A. Coelho M. A. PereiraMdo C. (2015). The potential effect of fluorinated compounds in the treatment of Alzheimer’s disease. *Curr. Pharm. Des.* 21 5725–5735. 10.2174/1381612821666150130120358 25633120

[B65] Gotz J. Schild A. Hoerndli F. Pennanen L. (2004). Amyloid-induced neurofibrillary tangle formation in Alzheimer’s disease: insight from transgenic mouse and tissue-culture models. *Int. J. Dev. Neurosci.* 22 453–465. 10.1016/j.ijdevneu.2004.07.013 15465275

[B66] Granzotto A. Zatta P. (2014). Resveratrol and Alzheimer’s disease: message in a bottle on red wine and cognition. *Front. Aging Neurosci.* 6:95 10.3389/fnagi.2014.00095 PMC4030174 24860502

[B67] Groothuis D. R. (2000). The blood-brain and blood-tumor barriers: a review of strategies for increasing drug delivery. *Neuro Oncol.* 2 45–59. 10.1093/neuonc/2.1.45 11302254 PMC1920694

[B68] Guo W. Li A. Jia Z. Yuan Y. Dai H. Li H. (2013). Transferrin modified PEG-PLA-resveratrol conjugates: in vitro and in vivo studies for glioma. *Eur. J. Pharmacol.* 718 41–47. 10.1016/j.ejphar.2013.09.034 24070814

[B69] Hald A. Lotharius J. (2005). Oxidative stress and inflammation in Parkinson’s disease: is there a causal link? *Exp. Neurol.* 193 279–290. 10.1016/j.expneurol.2005.01.013 15869932

[B70] Hao J. Zhao J. Zhang S. Tong T. Zhuang Q. Jin K. (2016). Fabrication of an ionic-sensitive in situ gel loaded with resveratrol nanosuspensions intended for direct nose-to-brain delivery. *Colloids Surf. B Biointerfaces* 147 376–386. 10.1016/j.colsurfb.2016.08.011 27566226

[B71] Haque S. Md S. Fazil M. Kumar M. Sahni J. K. Ali J. (2012). Venlafaxine loaded chitosan NPs for brain targeting: pharmacokinetic and pharmacodynamic evaluation. *Carbohydr. Polym.* 89 72–79. 10.1016/j.carbpol.2012.02.051 24750606

[B72] Hardy J. Selkoe D. (2002). The amyloid hypothesis of Alzheimer’s disease: progress and problems on the road to therapeutics. *Sci. Compass* 297 353–356. 10.1126/science.1072994 12130773

[B73] Hauser D. N. Hastings T. G. (2013). Mitochondrial dysfunction and oxidative stress in Parkinson’s disease and monogenic parkinsonism. *Neurobiol. Dis.* 51 35–42. 10.1016/j.nbd.2012.10.011 23064436 PMC3565564

[B74] Hayashibara T. Yamada Y. Nakayama S. Harasawa H. Tsuruda K. Sugahara K. (2002). Resveratrol induces downregulation in survivin expression and apoptosis in HTLV-1-infected cell lines: a prospective agent for adult T cell leukemia chemotherapy. *Nutr. Cancer* 44 193–201. 10.1207/S15327914NC4402_12 12734068

[B75] He X. Li Z. Rizak J. D. Wu S. Wang Z. He R. (2017). Resveratrol attenuates formaldehyde induced hyperphosphorylation of Tau protein and cytotoxicity in N2a cells. *Front. Neurosci.* 10:598. 10.3389/fnins.2016.00598 28197064 PMC5281604

[B76] Henchcliffe C. Beal M. F. (2008). Mitochondrial biology and oxidative stress in Parkinson disease pathogenesis. *Nat. Clin. Pract. Neurol.* 4 600–609. 10.1038/ncpneuro0924 18978800

[B77] Heneka M. T. O’Banion M. K. (2007). Inflammatory processes in Alzheimer’s disease. *J. Neuroimmunol.* 184 69–91. 10.1016/j.jneuroim.2006.11.017 17222916

[B78] Hirsch E. C. Jenner P. Przedborski S. (2013). Pathogenesis of Parkinson’s disease. *Mov. Disord.* 28 24–30. 10.1002/mds.25032 22927094

[B79] Householder K. T. DiPerna D. M. Chung E. P. Wohlleb G. M. Dhruv H. D. Berens M. E. (2015). Intravenous delivery of camptothecin-loaded PLGA nanoparticles for the treatment of intracranial glioma. *Int. J. Pharm.* 479 374–380. 10.1016/j.ijpharm.2015.01.002 25562639

[B80] Hu L. F. Lu M. Tiong C. X. Dawe G. S. Hu G. Bian J. S. (2010). Neuroprotective effects of hydrogen sulfide on Parkinson’s disease rat models. *Aging Cell* 9 135–146. 10.1111/j.1474-9726.2009.00543.x 20041858

[B81] Huang H. Lin H. Zhang X. Li J. (2012). Resveratrol reverses temozolomide resistance by downregulation of MGMT in T98G glioblastoma cells by the NF-kappaB-dependent pathway. *Oncol. Rep.* 27 2050–2056. 10.3892/or.2012.1715 22426504

[B82] Huang T. C. Lu K. T. Wo Y. Y. Wu Y. J. Yang Y. L. (2011). Resveratrol protects rats from Abeta-induced neurotoxicity by the reduction of iNOS expression and lipid peroxidation. *PLoS One* 6:e29102. 10.1371/journal.pone.0029102 22220203 PMC3248406

[B83] Jang J. H. Surh Y. J. (2003). Protective effect of resveratrol on β-amyloid-induced oxidative PC12 cell death. *Free Radic. Biol. Med.* 34 1100–1110. 10.1016/S0891-5849(03)00062-5 12684095

[B84] Jang M. Cai L. Udeani G. O. Slowing K. V. Thomas C. F. Beecher C. W. W. (1997). Cancer chemopreventive activity of resveratrol, a natural product derived from grapes. *Science* 275 218–220. 10.1126/science.275.5297.218 8985016

[B85] Jenner P. (2003). Oxidative stress in Parkinson’s disease. *Ann. Neurol.* 53(Suppl. 3), S26–S36. 10.1002/ana.10483 12666096

[B86] Jeon S. Y. Kwon S. H. Seong Y. H. Bae K. Hur J. M. Lee Y. Y. (2007). Beta-secretase (BACE1)-inhibiting stilbenoids from Smilax Rhizoma. *Phytomedicine* 14 403–408. 10.1016/j.phymed.2006.09.003 17084604

[B87] Jhang K. A. Park J. S. Kim H. S. Chong Y. H. (2017). Resveratrol ameliorates Tau hyperphosphorylation at Ser396 Site and oxidative damage in rat hippocampal slices exposed to vanadate: implication of ERK1/2 and GSK-3beta signaling cascades. *J. Agric. Food Chem.* 65 9626–9634. 10.1021/acs.jafc.7b03252 29022339

[B88] Jhaveri A. Deshpande P. Pattni B. Torchilin V. (2018). Transferrin-targeted, resveratrol-loaded liposomes for the treatment of glioblastoma. *J. Control. Release* 277 89–101. 10.1016/j.jconrel.2018.03.006 29522834 PMC5911193

[B89] Jiang H. Shang X. Wu H. Gautam S. C. Al-Holou S. Li C. (2009a). Resveratrol downregulates PI3K/Akt/mTOR signaling pathways in human U251 glioma cells. *J. Exp. Ther. Oncol.* 8 25–33. 19827268 PMC2833270

[B90] Jiang H. Shang X. Wu H. Huang G. Wang Y. Al-Holou S. (2009b). Combination treatment with resveratrol and sulforaphane induces apoptosis in human U251 glioma cells. *Neurochem. Res.* 35 152–161. 10.1007/s11064-009-0040-7 19685289 PMC2821708

[B91] Jiang H. Zhang L. Kuo J. Kuo K. Gautam S. C. Groc L. (2005). Resveratrol-induced apoptotic death in human U251 glioma cells. *Mol. Cancer Ther.* 4 554–561. 10.1158/1535-7163.MCT-04-0056 15827328

[B92] Jin F. Wu Q. Lu Y. F. Gong Q. H. Shi J. S. (2008). Neuroprotective effect of resveratrol on 6-OHDA-induced Parkinson’s disease in rats. *Eur. J. Pharmacol.* 600 78–82. 10.1016/j.ejphar.2008.10.005 18940189

[B93] Jose S. Anju S. S. Cinu T. A. Aleykutty N. A. Thomas S. Souto E. B. (2014). In vivo pharmacokinetics and biodistribution of resveratrol-loaded solid lipid nanoparticles for brain delivery. *Int. J. Pharm.* 474 6–13. 10.1016/j.ijpharm.2014.08.003 25102112

[B94] Kairisalo M. Bonomo A. Hyrskyluoto A. Mudo G. Belluardo N. Korhonen L. (2011). Resveratrol reduces oxidative stress and cell death and increases mitochondrial antioxidants and XIAP in PC6.3-cells. *Neurosci. Lett.* 488 263–266. 10.1016/j.neulet.2010.11.042 21094207

[B95] Karuppagounder S. S. Pinto J. T. Xu H. Chen H. L. Beal M. F. Gibson G. E. (2009). Dietary supplementation with resveratrol reduces plaque pathology in a transgenic model of Alzheimer’s disease. *Neurochem. Int.* 54 111–118. 10.1016/j.neuint.2008.10.008 19041676 PMC2892907

[B96] Khan I. Khan M. Umar M. N. Oh D. H. (2015). Nanobiotechnology and its applications in drug delivery system: a review. *IET Nanobiotechnol.* 9 396–400. 10.1049/iet-nbt.2014.0062 26647817

[B97] Kim D. Nguyen M. D. Dobbin M. M. Fischer A. Sananbenesi F. Rodgers J. T. (2007). SIRT1 deacetylase protects against neurodegeneration in models for Alzheimer’s disease and amyotrophic lateral sclerosis. *EMBO J.* 26 3169–3179. 10.1038/sj.emboj.7601758 17581637 PMC1914106

[B98] Kim Y. A. Lim S. Y. Rhee S. H. Park K. Y. Kim C. H. Choi B. T. (2006). Resveratrol inhibits inducible nitric oxide synthase and cyclooxygenase-2 expression in beta-amyloid-treated C6 glioma cells. *Int. J. Mol. Med.* 17 1069–1075. 16685418

[B99] Klafki H. W. Staufenbiel M. Kornhuber J. Wiltfang J. (2006). Therapeutic approaches to Alzheimer’s disease. *Brain* 129 2840–2855. 10.1093/brain/awl280 17018549

[B100] Kumar A. Naidu P. S. Seghal N. Padi S. S. V. (2007). Neuroprotective effects of resveratrol against intracerebroventricular colchicine-induced cognitive impairment and oxidative stress in rats. *Pharmacology* 79 17–26. 10.1159/000097511 17135773

[B101] Kurz A. Perneczky R. (2011). Amyloid clearance as a treatment target against Alzheimer’s disease. *J. Alzheimers Dis.* 24(Suppl. 2), 61–73. 10.3233/JAD-2011-102139 21422524

[B102] Kutuk O. Adli M. Poli G. Basaga H. (2004). Resveratrol protects against 4-HNE induced oxidative stress and apoptosis in Swiss 3T3 fibroblasts. *Biofactors* 20 1–10. 10.1002/biof.5520200101 15096656

[B103] Kwon K. J. Kim J. N. Kim M. K. Lee J. Ignarro L. J. Kim H. J. (2011). Melatonin synergistically increases resveratrol-induced heme oxygenase-1 expression through the inhibition of ubiquitin-dependent proteasome pathway: a possible role in neuroprotection. *J. Pineal Res.* 50 110–123. 10.1111/j.1600-079X.2010.00820.x 21073519

[B104] Ladiwala A. R. A. Lin J. C. Bale S. S. Marcelino-Cruz A. M. Bhattacharya M. Dordick J. S. (2010). Resveratrol selectively remodels soluble oligomers and fibrils of amyloid Aβ into off-pathway conformers. *J. Biol. Chem.* 285 24228–24237. 10.1074/jbc.M110.133108 20511235 PMC2911349

[B105] Lee M. K. Kang S. J. Poncz M. Song K. J. Park K. S. (2007). Resveratrol protects SH-SY5Y neuroblastoma cells from apoptosis induced by dopamine. *Exp. Mol. Med.* 39 376–384. 10.1038/emm.2007.42 17603292

[B106] Lee V. M. Goedert M. Trojanowski J. Q. (2001). Neurodegenerative tauopathies. *Annu. Rev. Neurosci.* 24 1121–1159. 10.1146/annurev.neuro.24.1.1121 11520930

[B107] Lee V. M. Trojanowski J. Q. (2006). Progress from Alzheimer’s tangles to pathological Tau points towards more effective therapies now. *J. Alzheimers Dis.* 9 257–262. 10.3233/JAD-2006-9S328 16914864

[B108] Li F. Gong Q. Dong H. Shi J. (2012). Resveratrol, a neuroprotective supplement for Alzheimer’s disease. *Curr. Pharm. Des.* 18 27–33. 10.2174/138161212798919075 22211686

[B109] Li H. Liu Y. Jiao Y. Guo A. Xu X. Qu X. (2016). Resveratrol sensitizes glioblastoma-initiating cells to temozolomide by inducing cell apoptosis and promoting differentiation. *Oncol. Rep.* 35 343–351. 10.3892/or.2015.4346 26498391

[B110] Li X. Tsibouklis J. Weng T. Zhang B. Yin G. Feng G. (2017). Nano carriers for drug transport across the blood–brain barrier. *J. Drug Target.* 25 17–28. 10.1080/1061186X.2016.1184272 27126681

[B111] Li X. Y. Zhao Y. Sun M. G. Shi J. F. Ju R. J. Zhang C. X. (2014). Multifunctional liposomes loaded with paclitaxel and artemether for treatment of invasive brain glioma. *Biomaterials* 35 5591–5604. 10.1016/j.biomaterials.2014.03.049 24726749

[B112] Liebner S. Czupalla C. J. Wolburg H. (2011). Current concepts of blood-brain barrier development. *Int. J. Dev. Biol.* 55 467–476. 10.1387/ijdb.103224sl 21769778

[B113] Lin C. J. Lee C. C. Shih Y. L. Lin T. Y. Wang S. H. Lin Y. F. (2012). Resveratrol enhances the therapeutic effect of temozolomide against malignant glioma in vitro and in vivo by inhibiting autophagy. *Free Radic. Biol. Med.* 52 377–391. 10.1016/j.freeradbiomed.2011.10.487 22094224

[B114] Liu B. Hong J. S. (2003). Role of microglia in inflammation-mediated neurodegenerative diseases: mechanisms and strategies for therapeutic intervention. *J. Pharmacol. Exp. Ther.* 304 1–7. 10.1124/jpet.102.035048 12490568

[B115] Loureiro J. Andrade S. Duarte A. Neves A. Queiroz J. Nunes C. (2017). Resveratrol and grape extract-loaded solid lipid nanoparticles for the treatment of Alzheimer’s disease. *Molecules* 22:E277. 10.3390/molecules22020277 28208831 PMC6155722

[B116] Loureiro J. A. Coelho M. A. N. Rocha S. Pereira M. D. C. (2012). “Design of potential therapeutic peptides and carriers to inhibit amyloid β peptide aggregation,” in *Proceedings of the 2nd Portuguese Meeting in Bioengineering (ENBENG)*, (Coimbra: IEEE), 1–4. 10.1109/ENBENG.2012.6331364

[B117] Loureiro J. A. Crespo R. Börner H. Martins P. M. Rocha F. A. Coelho M. (2014a). Fluorinated beta-sheet breaker peptides. *J. Mater. Chem. B* 2 2259–2264. 10.1002/bip.22670 32261713

[B118] Loureiro J. A. Gomes B. Coelho M. A. do Carmo Pereira M. Rocha S. (2014b). Targeting nanoparticles across the blood-brain barrier with monoclonal antibodies. *Nanomedicine* 9 709–722. 10.2217/nnm.14.27 24827845

[B119] Loureiro J. A. Gomes B. Coelho M. A. Pereira M. D. C. Rocha S. (2013a). “Immunoliposomes for Alzheimer’s disease therapy,” in *Proceedings of the 3rd Portuguese Meeting in Bioengineering (ENBENG)*, (Braga: IEEE), 1–3. 10.1109/ENBENG.2013.6518392

[B120] Loureiro J. A. Rocha S. Pereira Mdo C. (2013b). Charged surfactants induce a non-fibrillar aggregation pathway of amyloid-beta peptide. *J. Pept. Sci.* 19 581–587. 10.1002/psc.2535 23922329

[B121] Loureiro J. A. Gomes B. Coelho M. A. N. do Carmo Pereira and Rocha S. (2015a). Immunoliposomes doubly targeted to transferrin receptor and to α-synuclein. *Future Sci. OA* 1:FSO71. 10.4155/fso.15.71 28031922 PMC5137902

[B122] Loureiro J. A. Gomes B. Fricker G. Cardoso I. Ribeiro C. A. Gaiteiro C. (2015b). Dual ligand immunoliposomes for drug delivery to the brain. *Colloids Surf. B Biointerfaces* 134 213–219. 10.1016/j.colsurfb.2015.06.067 26204501

[B123] Loureiro J. A. Gomes B. Fricker G. Coelho M. A. N. Rocha S. Pereira M. C. (2016). Cellular uptake of PLGA nanoparticles targeted with anti-amyloid and anti-transferrin receptor antibodies for Alzheimer’s disease treatment. *Colloids Surf. B Biointerfaces* 145 8–13. 10.1016/j.colsurfb.2016.04.041 27131092

[B124] Lu X. Ji C. Xu H. Li X. Ding H. Ye M. (2009). Resveratrol-loaded polymeric micelles protect cells from Abeta-induced oxidative stress. *Int. J. Pharm.* 375 89–96. 10.1016/j.ijpharm.2009.03.021 19481694

[B125] Lu X. Ma L. Ruan L. Kong Y. Mou H. Zhang Z. (2010). Resveratrol differentially modulates inflammatory responses of microglia and astrocytes. *J. Neuroinflammation* 7:46. 10.1186/1742-2094-7-46 20712904 PMC2936301

[B126] Lu X. Xu H. Sun B. Zhu Z. Zheng D. Li X. (2013). Enhanced neuroprotective effects of resveratrol delivered by nanoparticles on hydrogen peroxide-induced oxidative stress in rat cortical cell culture. *Mol. Pharm.* 10 2045–2053. 10.1021/mp400056c 23534345

[B127] Ma T. Tan M.-S. Yu J.-T. Tan L. (2014). Resveratrol as a therapeutic agent for Alzheimer’s disease. *Biomed Res. Int.* 2014:350516. 10.1155/2014/350516 25525597 PMC4261550

[B128] Mallick S. Gandhi A. K. Rath G. K. (2015). Therapeutic approach beyond conventional temozolomide for newly diagnosed glioblastoma: review of the present evidence and future direction. *Indian J. Med. Paediatr. Oncol.* 36 229–237. 10.4103/0971-5851.171543 26811592 PMC4711221

[B129] Marambaud P. Zhao H. Davies P. (2005). Resveratrol promotes clearance of Alzheimer’s disease amyloid-beta peptides. *J. Biol. Chem.* 280 37377–37382. 10.1074/jbc.M508246200 16162502

[B130] Martin I. (2017). Resveratrol for Alzheimer’s disease. *Sci. Transl. Med.* 1403 142–149. 10.1126/scitranslmed.aam6055 28148845

[B131] Masserini M. (2013). Nanoparticles for brain drug delivery. *ISRN Biochem.* 2013:238428. 10.1155/2013/238428 25937958 PMC4392984

[B132] Mawuenyega K. G. Sigurdson W. Ovod V. Munsell L. Kasten T. Morris J. C. (2010). Decreased clearance of CNS beta-amyloid in Alzheimer’s disease. *Science* 330:1774. 10.1126/science.1197623 21148344 PMC3073454

[B133] Meraz-Ríos M. León K. Campos-Penã V. Anda-Hernández M. Mena-López R. (2010). Tau oligomers and aggregation in Alzheimer’s disease. *J. Neurochem.* 112 1353–1367. 10.1111/j.1471-4159.2009.06511.x 19943854

[B134] Milne J. C. Denu J. M. (2008). The Sirtuin family: therapeutic targets to treat diseases of aging. *Curr. Opin. Chem. Biol.* 12 11–17. 10.1016/j.cbpa.2008.01.019 18282481

[B135] Min S. W. Cho S. H. Zhou Y. Schroeder S. Haroutunian V. Seeley W. W. (2010). Acetylation of Tau inhibits its degradation and contributes to tauopathy. *Neuron* 67 953–966. 10.1016/j.neuron.2010.08.044 20869593 PMC3035103

[B136] Mishra R. Sellin D. Radovan D. Gohlke A. Winter R. (2009). Inhibiting islet amyloid polypeptide fibril formation by the red wine compound resveratrol. *Chembiochem* 10 445–449. 10.1002/cbic.200800762 19165839

[B137] Mittal D. Ali A. Md S. Baboota S. Sahni J. K. Ali J. (2014). Insights into direct nose to brain delivery: current status and future perspective. *Drug Deliv.* 21 75–86. 10.3109/10717544.2013.838713 24102636

[B138] Mokni M. Elkahoui S. Limam F. Amri M. Aouani E. (2007). Effect of resveratrol on antioxidant enzyme activities in the brain of healthy rat. *Neurochem. Res.* 32 981–987. 10.1007/s11064-006-9255-z 17401679

[B139] Moussa C. Hebron M. Huang X. Ahn J. Rissman R. A. Aisen P. S. (2017). Resveratrol regulates neuro-inflammation and induces adaptive immunity in Alzheimer’s disease. *J. Neuroinflammation* 14:1. 10.1186/s12974-016-0779-0 28086917 PMC5234138

[B140] Murakami K. Irie K. Ohigashi H. Hara H. Nagao M. Shimizu T. (2005). Formation and stabilization model of the 42-mer Abeta radical: implications for the long-lasting oxidative stress in Alzheimer’s disease. *J. Am. Chem. Soc.* 127 15168–15174. 10.1021/ja054041c 16248658

[B141] Neves A. R. Lucio M. Lima J. L. Reis S. (2012). Resveratrol in medicinal chemistry: a critical review of its pharmacokinetics, drug-delivery, and membrane interactions. *Curr. Med. Chem.* 19 1663–1681. 10.2174/092986712799945085 22257059

[B142] Neves A. R. Queiroz J. F. Reis S. (2016). Brain-targeted delivery of resveratrol using solid lipid nanoparticles functionalized with apolipoprotein E. *J. Nanobiotechnol.* 14:27. 10.1186/s12951-016-0177-x 27061902 PMC4826547

[B143] Nicolini G. Rigolio R. Miloso M. Bertelli A. A. Tredici G. (2001). Anti-apoptotic effect of trans-resveratrol on paclitaxel-induced apoptosis in the human neuroblastoma SH-SY5Y cell line. *Neurosci. Lett.* 302 41–44. 10.1016/S0304-3940(01)01654-8 11278107

[B144] Olanow C. W. Agid Y. Mizuno Y. Albanese A. Bonuccelli U. Damier P. (2004). Levodopa in the treatment of Parkinson’s disease: current controversies. *Mov. Disord.* 19 997–1005. 10.1002/mds.20243 15372588

[B145] Olanow C. W. Tatton W. G. (1999). Etiology and pathogenesis of Parkinson’s disease. *Annu. Rev. Neurosci.* 22 123–144. 10.1146/annurev.neuro.22.1.123 10202534

[B146] Palle S. Neerati P. (2018). Improved neuroprotective effect of resveratrol nanoparticles as evinced by abrogation of rotenone-induced behavioral deficits and oxidative and mitochondrial dysfunctions in rat model of Parkinson’s disease. *Naunyn Schmiedebergs Arch. Pharmacol.* 391 445–453. 10.1007/s00210-018-1474-8 29411055

[B147] Pangeni R. Sharma S. Mustafa G. Ali J. Baboota S. (2014). Vitamin E loaded resveratrol nanoemulsion for brain targeting for the treatment of Parkinson’s disease by reducing oxidative stress. *Nanotechnology* 25:485102. 10.1088/0957-4484/25/48/485102 25392203

[B148] Pardeshi C. V. Belgamwar V. S. Tekade A. R. Surana S. J. (2013a). Novel surface modified polymer-lipid hybrid nanoparticles as intranasal carriers for ropinirole hydrochloride: in vitro, ex vivo and in vivo pharmacodynamic evaluation. *J. Mater. Sci. Mater. Med.* 24 2101–2115. 10.1007/s10856-013-4965-7 23728521

[B149] Pardeshi C. V. Rajput P. V. Belgamwar V. S. Tekade A. R. Surana S. J. (2013b). Novel surface modified solid lipid nanoparticles as intranasal carriers for ropinirole hydrochloride: application of factorial design approach. *Drug Deliv.* 20 47–56. 10.3109/10717544.2012.752421 23311653

[B150] Pardridge W. M. (2009). Alzheimer’s disease drug development and the problem of the blood-brain barrier. *Alzheimers Dement.* 5 427–432. 10.1016/j.jalz.2009.06.003 19751922 PMC2756824

[B151] Pasinetti G. M. Wang J. Ho L. Zhao W. Dubner L. (2015). Roles of resveratrol and other grape-derived polyphenols in Alzheimer’s disease prevention and treatment. *Biochim. Biophys. Acta* 1852 1202–1208. 10.1016/j.bbadis.2014.10.006 25315300 PMC4380832

[B152] Patil S. P. Tran N. Geekiyanage H. Liu L. Chan C. (2013). Curcumin-induced upregulation of the anti-Tau cochaperone BAG2 in primary rat cortical neurons. *Neurosci. Lett.* 554 121–125. 10.1016/j.neulet.2013.09.008 24035895 PMC3825752

[B153] Pehlivan S. B. (2013). Nanotechnology-based drug delivery systems for targeting, imaging and diagnosis of neurodegenerative diseases. *Pharm. Res.* 30 2499–2511. 10.1007/s11095-013-1156-7 23959851

[B154] Peres I. Rocha S. Loureiro J. A. do Carmo Pereira M. Ivanova G. Coelho M. (2012). Carbohydrate particles as protein carriers and scaffolds: physico-chemical characterization and collagen stability. *J. Nanopart. Res.* 14:1144 10.1007/s11051-012-1144-6

[B155] Peterson L. J. Flood P. M. (2012). Oxidative stress and microglial cells in Parkinson’s disease. *Mediators Inflamm.* 2012:401264. 10.1155/2012/401264 22544998 PMC3321615

[B156] Pirtoli L. Belmonte G. Toscano M. Tini P. Miracco C. (2016). Cyclin D1 Co-localizes with Beclin-1 in glioblastoma recurrences: a clue to a therapy-induced, autophagy-mediated degradative mechanism? *Anticancer Res.* 36 4057–4062. 27466513

[B157] Porquet D. Casadesus G. Bayod S. Vicente A. Canudas A. M. Vilaplana J. (2013). Dietary resveratrol prevents Alzheimer’s markers and increases life span in SAMP8. *Age* 35 1851–1865. 10.1007/s11357-012-9489-4 23129026 PMC3776096

[B158] Porquet D. Grinan-Ferre C. Ferrer I. Camins A. Sanfeliu C. Del Valle J. (2014). Neuroprotective role of trans-resveratrol in a murine model of familial Alzheimer’s disease. *J. Alzheimers Dis.* 42 1209–1220. 10.3233/JAD-140444 25024312

[B159] Ramalho M. J. Coelho M. A. N. Pereira M. C. (2018a). “Chapter 18 - Nanocarriers for the delivery of temozolomide in the treatment of glioblastoma: a review,” in *Design and Development of New Nanocarriers*, ed. Grumezescu A. M. (New York, NY: William Andrew Publishing), 687–722. 10.1016/B978-0-12-813627-0.00018-1

[B160] Ramalho M. J. Sevin E. Gosselet F. Lima J. Coelho M. A. N. Loureiro J. A. (2018b). Receptor-mediated PLGA nanoparticles for glioblastoma multiforme treatment. *Int. J. Pharm.* 545 84–92. 10.1016/j.ijpharm.2018.04.062 29715532

[B161] Ramalho M. J. Loureiro J. A. Gomes B. Frasco M. F. Coelho M. A. Pereira M. C. (2015a). PLGA nanoparticles as a platform for vitamin D-based cancer therapy. *Beilstein J. Nanotechnol.* 6 1306–1318. 10.3762/bjnano.6.135 26199834 PMC4505177

[B162] Ramalho M. J. Loureiro J. A. Gomes B. Frasco M. F. Coelho M. A. N. Pereira M. C. (2015b). “PLGA nanoparticles for calcitriol delivery,” in *Proceedings of the 4th Portuguese Meeting on Bioengineering (ENBENG)*, (Porto: IEEE), 1–6. 10.1109/ENBENG.2015.7088884

[B163] Ramalho M. J. Pereira M. C. (2016). Preparation and characterization of polymeric nanoparticles: an interdisciplinary experiment. *J. Chem. Educ.* 93 1446–1451. 10.1021/acs.jchemed.5b00837

[B164] Rauf A. Imran M. Butt M. S. Nadeem M. Peters D. G. Mubarak M. S. (2018). Resveratrol as an anti-cancer agent: a review. *Crit. Rev. Food Sci. Nutr.* 58 1428–1447. 10.1080/10408398.2016.1263597 28001084

[B165] Rauf A. Imran M. Suleria H. A. R. Ahmad B. Peters D. G. Mubarak M. S. (2017). A comprehensive review of the health perspectives of resveratrol. *Food Funct.* 8 4284–4305. 10.1039/c7fo01300k 29044265

[B166] Reddy P. H. Beal M. F. (2005). Are mitochondria critical in the pathogenesis of Alzheimer’s disease? *Brain Res. Brain Res. Rev.* 49 618–632. 10.1016/j.brainresrev.2005.03.004 16269322

[B167] Rege S. D. Geetha T. Broderick T. L. Babu J. R. (2015). Resveratrol protects β amyloid-induced oxidative damage and memory associated proteins in H19-7 hippocampal neuronal cells. *Curr. Alzheimer Res.* 12 147–156. 10.2174/1567205012666150204130009 25654502

[B168] Rege S. D. Geetha T. Griffin G. D. Broderick T. L. Babu J. R. (2014). Neuroprotective effects of resveratrol in Alzheimer disease pathology. *Front. Aging Neurosci.* 6:218 10.3389/fnagi.2014.00218 PMC4161050 25309423

[B169] Ribeiro C. A. Saraiva M. J. Cardoso I. (2012). Stability of the transthyretin molecule as a key factor in the interaction with A-beta peptide - relevance in Alzheimer’s disease. *PLoS One* 7:e45368. 10.1371/journal.pone.0045368 23028965 PMC3444465

[B170] Rivlin N. Brosh R. Oren M. Rotter V. (2011). Mutations in the p53 tumor suppressor gene: important milestones at the various steps of tumorigenesis. *Genes Cancer* 2 466–474. 10.1177/1947601911408889 21779514 PMC3135636

[B171] Rocha S. Loureiro J. A. Brezesinski G. Pereira Mdo C. (2012). Peptide-surfactant interactions: consequences for the amyloid-beta structure. *Biochem. Biophys. Res. Commun.* 420 136–140. 10.1016/j.bbrc.2012.02.129 22405768

[B172] Rushworth J. V. Griffiths H. H. Watt N. T. Hooper N. M. (2013). Prion protein-mediated toxicity of amyloid-beta oligomers requires lipid rafts and the transmembrane LRP1. *J. Biol. Chem.* 288 8935–8951. 10.1074/jbc.M112.400358 23386614 PMC3610967

[B173] Ryu J. Ku B. M. Lee Y. K. Jeong J. Y. Kang S. Choi J. (2011). Resveratrol reduces TNF-α-induced U373MG human glioma cell invasion through regulating NF-κB activation and uPA/uPAR expression. *Anticancer Res.* 31 4223–4230. 22199285

[B174] Sadhukhan P. Saha S. Dutta S. Mahalanobish S. Sil P. C. (2018). Nutraceuticals: an emerging therapeutic approach against the pathogenesis of Alzheimer’s disease. *Pharmacol. Res.* 129 100–114. 10.1016/j.phrs.2017.11.028 29183770

[B175] Sagare A. P. Bell R. D. Zlokovic B. V. (2012). Neurovascular dysfunction and faulty amyloid beta-peptide clearance in Alzheimer disease. *Cold Spring Harbor. Perspect. Med.* 2:a011452. 10.1101/cshperspect.a011452 23028132 PMC3475405

[B176] Saha A. Sarkar C. Singh S. P. Zhang Z. Munasinghe J. Peng S. (2012). The blood-brain barrier is disrupted in a mouse model of infantile neuronal ceroid lipofuscinosis: amelioration by resveratrol. *Hum. Mol. Genet.* 21 2233–2244. 10.1093/hmg/dds038 22331300 PMC3335311

[B177] Saravanan K. S. Sindhu K. M. Mohanakumar K. P. (2005). Acute intranigral infusion of rotenone in rats causes progressive biochemical lesions in the striatum similar to Parkinson’s disease. *Brain Res.* 1049 147–155. 10.1016/j.brainres.2005.04.051 15936733

[B178] Sathya M. Moorthi P. Premkumar P. Kandasamy M. Jayachandran K. S. Anusuyadevi M. (2017). Resveratrol intervenes cholesterol- and isoprenoid-mediated amyloidogenic processing of AbetaPP in familial Alzheimer’s disease. *J. Alzheimers Dis.* 60 S3–S23. 10.3233/JAD-161034 28059793

[B179] Savaskan E. Olivieri G. Meier F. Seifritz E. Wirz-Justice A. Müller-Spahn F. (2003). Red wine ingredient resveratrol protects from β-amyloid neurotoxicity. *Gerontology* 49 380–383. 10.1159/000073766 14624067

[B180] Schapira A. H. (2009). Neurobiology and treatment of Parkinson’s disease. *Trends Pharmacol. Sci.* 30 41–47. 10.1016/j.tips.2008.10.005 19042040

[B181] Schapira A. H. Bezard E. Brotchie J. Calon F. Collingridge G. L. Ferger B. (2006). Novel pharmacological targets for the treatment of Parkinson’s disease. *Nat. Rev. Drug Discov.* 5 845–854. 10.1038/nrd2087 17016425

[B182] Schneider A. Mandelkow E. (2008). Tau-based treatment strategies in neurodegenerative diseases. *Neurotherapeutics* 5 443–457. 10.1016/j.nurt.2008.05.006 18625456 PMC5084246

[B183] Schroeder U. Sommerfeld P. Ulrich S. Sabel B. A. (1998). Nanoparticle technology for delivery of drugs across the blood-brain barrier. *J. Pharm. Sci.* 87 1305–1307. 10.1021/js980084y 9811481

[B184] Schweiger S. Matthes F. Posey K. Kickstein E. Weber S. Hettich M. M. (2017). Resveratrol induces dephosphorylation of Tau by interfering with the MID1-PP2A complex. *Sci. Rep.* 7:13753. 10.1038/s41598-017-12974-4 29062069 PMC5653760

[B185] Scuderi C. Stecca C. Bronzuoli M. R. Rotili D. Valente S. Mai A. (2014). Sirtuin modulators control reactive gliosis in an in vitro model of Alzheimer’s disease. *Front. Pharmacol.* 5:89 10.3389/fphar.2014.00089 PMC4027795 24860504

[B186] Selkoe D. J. (2001). Alzheimer’s disease: genes, proteins, and therapy. *Physiol. Rev.* 81 741–766. 10.1152/physrev.2001.81.2.741 11274343

[B187] Seo E. J. Fischer N. Efferth T. (2018). Phytochemicals as inhibitors of NF-κB for treatment of Alzheimer’s disease. *Pharmacol. Res.* 129 262–273. 10.1016/j.phrs.2017.11.030 29179999

[B188] Shao J. Li X. Lu X. Jiang C. Hu Y. Li Q. (2009). Enhanced growth inhibition effect of Resveratrol incorporated into biodegradable nanoparticles against glioma cells is mediated by the induction of intracellular reactive oxygen species levels. *Colloids Surf. B Biointerfaces* 72 40–47. 10.1016/j.colsurfb.2009.03.010 19395246

[B189] Shen Y. Cao B. Snyder N. R. Woeppel K. M. Eles J. R. Cui X. T. (2018). ROS responsive resveratrol delivery from LDLR peptide conjugated PLA-coated mesoporous silica nanoparticles across the blood–brain barrier. *J. Nanobiotechnol.* 16:13. 10.1186/s12951-018-0340-7 29433522 PMC5810018

[B190] Sinclair D. A. Guarente L. (2014). Small-molecule allosteric activators of sirtuins. *Annu. Rev. Pharmacol. Toxicol.* 54 363–380. 10.1146/annurev-pharmtox-010611-134657 24160699 PMC4018738

[B191] Slevin M. Ahmed N. Wang Q. McDowell G. Badimon L. (2012). Unique vascular protective properties of natural products: supplements or future main-line drugs with significant anti-atherosclerotic potential? *Vasc. Cell* 4:9. 10.1186/2045-824X-4-9 22546170 PMC3508621

[B192] Small S. Duff K. (2008). Linking Ab and Tau in late-onset Alzheimer’s disease: a dual pathway hypothesis. *Neuron* 60 534–542. 10.1016/j.neuron.2008.11.007 19038212 PMC2692134

[B193] Smith S. J. Ward J. H. Tan C. Grundy R. G. Rahman R. (2015). Endothelial-like malignant glioma cells in dynamic three dimensional culture identifies a role for VEGF and FGFR in a tumor-derived angiogenic response. *Oncotarget* 6 22191–22205. 10.18632/oncotarget.4339 26203665 PMC4673156

[B194] Sonmez U. Sonmez A. Erbil G. Tekmen I. Baykara B. (2007). Neuroprotective effects of resveratrol against traumatic brain injury in immature rats. *Neurosci. Lett.* 420 133–137. 10.1016/j.neulet.2007.04.070 17531385

[B195] Summerlin N. Soo E. Thakur S. Qu Z. Jambhrunkar S. Popat A. (2015). Resveratrol nanoformulations: challenges and opportunities. *Int. J. Pharm.* 479 282–290. 10.1016/j.ijpharm.2015.01.003 25572692

[B196] Swarnkar S. Singh S. Sharma S. Mathur R. Patro I. K. Nath C. (2011). Rotenone induced neurotoxicity in rat brain areas: a histopathological study. *Neurosci. Lett.* 501 123–127. 10.1016/j.neulet.2011.03.036 21435374

[B197] Szabo G. (2009). A glass of red wine to improve mitochondrial biogenesis? Novel mechanisms of resveratrol. *Am. J. Physiol. Heart Circ. Physiol.* 297 H8–H9. 10.1152/ajpheart.00471.2009 19465547

[B198] Takuma K. Fang F. Zhang W. Yan S. Fukuzaki E. Du H. (2009). RAGE-mediated signaling contributes to intraneuronal transport of amyloid-beta and neuronal dysfunction. *Proc. Natl. Acad. Sci. U.S.A.* 106 20021–20026. 10.1073/pnas.0905686106 19901339 PMC2785285

[B199] Tamagno E. Bardini P. Obbili A. Vitali A. Borghi R. Zaccheo D. (2002). Oxidative stress increases expression and activity of BACE in NT2 neurons. *Neurobiol. Dis.* 10 279–288. 10.1006/nbdi.2002.0515 12270690

[B200] Tansey M. G. McCoy M. K. Frank-Cannon T. C. (2007). Neuroinflammatory mechanisms in Parkinson’s disease: potential environmental triggers, pathways, and targets for early therapeutic intervention. *Exp. Neurol.* 208 1–25. 10.1016/j.expneurol.2007.07.004 17720159 PMC3707134

[B201] Tanzi R. E. Moir R. D. Wagner S. L. (2004). Clearance of Alzheimer’s Abeta peptide: the many roads to perdition. *Neuron* 43 605–608. 15339642 10.1016/j.neuron.2004.08.024

[B202] Tapias V. Cannon J. R. Greenamyre J. T. (2014). Pomegranate juice exacerbates oxidative stress and nigrostriatal degeneration in Parkinson’s disease. *Neurobiol. Aging* 35 1162–1176. 10.1016/j.neurobiolaging.2013.10.077 24315037 PMC3946191

[B203] Tellone E. Galtieri A. Russo A. Giardina B. Ficarra S. (2015). Resveratrol: a focus on several neurodegenerative diseases. *Oxid. Med. Cell. Longev.* 2015:392169. 10.1155/2015/392169 26180587 PMC4477222

[B204] Trotta V. Pavan B. Ferraro L. Beggiato S. Traini D. Des Reis L. G. (2018). Brain targeting of resveratrol by nasal administration of chitosan-coated lipid microparticles. *Eur. J. Pharm. Biopharm.* 127 250–259. 10.1016/j.ejpb.2018.02.010 29486302

[B205] Tseng S. H. Lin S. M. Chen J. C. Su Y. H. Huang H. Y. Chen C. K. (2004). Resveratrol suppresses the angiogenesis and tumor growth of gliomas in rats. *Clin. Cancer Res.* 10 2190–2202. 10.1158/1078-0432.CCR-03-0105 15041740

[B206] Vafadari B. Salamian A. Kaczmarek L. (2016). MMP-9 in translation: from molecule to brain physiology, pathology, and therapy. *J. Neurochem.* 139(Suppl. 2), 91–114. 10.1111/jnc.13415 26525923

[B207] Varoni E. M. Lo Faro A. F. Sharifi-Rad J. Iriti M. (2016). Anticancer molecular mechanisms of resveratrol. *Front. Nutr.* 3:8. 10.3389/fnut.2016.00008 27148534 PMC4828556

[B208] Vijayakumar M. R. Kosuru R. Singh S. K. Prasad C. B. Narayan G. Muthu M. S. (2016a). Resveratrol loaded PLGA:d-[small alpha]-tocopheryl polyethylene glycol 1000 succinate blend nanoparticles for brain cancer therapy. *RSC Adv.* 6 74254–74268. 10.1039/C6RA15408E

[B209] Vijayakumar M. R. Kosuru R. Vuddanda P. R. Singh S. K. Singh S. (2016b). Trans resveratrol loaded DSPE PEG 2000 coated liposomes: an evidence for prolonged systemic circulation and passive brain targeting. *J. Drug Deliv. Sci. Technol.* 33 125–135. 10.1016/j.jddst.2016.02.009

[B210] Vijayakumar M. R. Kumari L. Patel K. K. Vuddanda P. R. Vajanthri K. Y. Mahto S. K. (2016c). Intravenous administration of trans-resveratrol-loaded TPGS-coated solid lipid nanoparticles for prolonged systemic circulation, passive brain targeting and improved in vitro cytotoxicity against C6 glioma cell lines. *RSC Adv.* 6 50336–50348. 10.1039/C6RA10777J

[B211] Vijayakumar M. R. Vajanthri K. Y. Balavigneswaran C. K. Mahto S. K. Mishra N. Muthu M. S. (2016d). Pharmacokinetics, biodistribution, in vitro cytotoxicity and biocompatibility of Vitamin E TPGS coated trans resveratrol liposomes. *Colloids Surf. B Biointerfaces* 145 479–491. 10.1016/j.colsurfb.2016.05.037 27236510

[B212] Vingtdeux V. Davies P. Dickson D. W. Marambaud P. (2011). AMPK is abnormally activated in tangle- and pre-tangle-bearing neurons in Alzheimer’s disease and other tauopathies. *Acta Neuropathol.* 121 337–349. 10.1007/s00401-010-0759-x 20957377 PMC3060560

[B213] Vingtdeux V. Dreses-Werringloer U. Zhao H. Davies P. Marambaud P. (2008). Therapeutic potential of resveratrol in Alzheimer’s disease. *BMC Neurosci.* 9(Suppl. 2):S6. 10.1186/1471-2202-9-S2-S6 19090994 PMC2604890

[B214] Vingtdeux V. Giliberto L. Zhao H. Chandakkar P. Wu Q. Simon J. E. (2010). AMP-activated protein kinase signaling activation by resveratrol modulates amyloid-beta peptide metabolism. *J. Biol. Chem.* 285 9100–9113. 10.1074/jbc.M109.060061 20080969 PMC2838330

[B215] Virgili M. Contestabile A. (2000). Partial neuroprotection of in vivo excitotoxic brain damage by chronic administration of the red wine antioxidant agent, trans-resveratrol in rats. *Neurosci. Lett.* 281 123–126. 10.1016/S0304-3940(00)00820-X 10704758

[B216] Walsh D. M. Selkoe D. J. (2007). A beta oligomers - a decade of discovery. *J. Neurochem.* 101 1172–1184. 10.1111/j.1471-4159.2006.04426.x 17286590

[B217] Wang F. Chatterjee S. (2017). Dominant carbons in trans- and cis-resveratrol isomerization. *J. Phys. Chem. B* 121 4745–4755. 10.1021/acs.jpcb.7b02115 28402662

[B218] Wang G. Dai F. Yu K. Jia Z. Zhang A. Huang Q. (2015). Resveratrol inhibits glioma cell growth via targeting oncogenic microRNAs and multiple signaling pathways. *Int. J. Oncol.* 46 1739–1747. 10.3892/ijo.2015.2863 25646654

[B219] Wang H. Jiang T. Li W. Gao N. Zhang T. (2018). Resveratrol attenuates oxidative damage through activating mitophagy in an in vitro model of Alzheimer’s disease. *Toxicol. Lett.* 282 100–108. 10.1016/j.toxlet.2017.10.021 29097221

[B220] Wang H. Liu J. Gao G. Wu X. Wang X. Yang H. (2016). Protection effect of piperine and piperlonguminine from *Piper longum* L. alkaloids against rotenone-induced neuronal injury. *Brain Res.* 1639 214–227. 10.1016/j.brainres.2015.07.029 26232071

[B221] Wang S. Wang Z. Yang S. Yin T. Zhang Y. Qin Y. (2017). Tissue distribution of *trans*-resveratrol and its metabolites after oral administration in human eyes. *J. Ophthalmol.* 2017:4052094. 10.1155/2017/4052094 28409021 PMC5377058

[B222] Wang Y. Xu H. Fu Q. Ma R. Xiang J. (2011). Protective effect of resveratrol derived from *Polygonum cuspidatum* and its liposomal form on nigral cells in parkinsonian rats. *J. Neurol. Sci.* 304 29–34. 10.1016/j.jns.2011.02.025 21376343

[B223] Wenk G. L. (2003). Neuropathologic changes in Alzheimer’s disease. *J. Clin. Psychiatry* 64(Suppl. 9), 7–10. 12934968

[B224] Wilcock G. K. Esiri M. M. (1982). Plaques, tangles and dementia. A quantitative study. *J. Neurol. Sci.* 56 343–356. 10.1016/0022-510X(82)90155-1 7175555

[B225] Wohlfart S. Gelperina S. Kreuter J. (2012). Transport of drugs across the blood-brain barrier by nanoparticles. *J. Control. Release* 161 264–273. 10.1016/j.jconrel.2011.08.017 21872624

[B226] Xin Y. Liu T. Yang C. (2016). Development of PLGA-lipid nanoparticles with covalently conjugated indocyanine green as a versatile nanoplatform for tumor-targeted imaging and drug delivery. *Int. J. Nanomedicine* 11 5807–5821. 10.2147/IJN.S119999 27853366 PMC5104302

[B227] Xu H. Jia F. Singh P. K. Ruan S. Zhang H. Li X. (2017). Synergistic anti-glioma effect of a coloaded nano-drug delivery system. *Int. J. Nanomedicine* 12 29–40. 10.2147/IJN.S116367 28031711 PMC5179207

[B228] Yang T. Wang L. Zhu M. Zhang L. Yan L. (2015). Properties and molecular mechanisms of resveratrol: a review. *Pharmazie* 70 501–506. 26380517

[B229] Yao H. Wang K. Wang Y. Wang S. Li J. Lou J. (2015). Enhanced blood–brain barrier penetration and glioma therapy mediated by a new peptide modified gene delivery system. *Biomaterials* 37 345–352. 10.1016/j.biomaterials.2014.10.034 25453963

[B230] Yi-Ping Y. Yuh-Lih C. Pin H. Guang-Yuh I. C. Ling-Ming T. Shih-Hwa C. (2012). Resveratrol suppresses tumorigenicity and enhances radiosensitivity in primary glioblastoma tumor initiating cells by inhibiting the STAT3 axis. *J. Cell. Physiol.* 227 976–993. 10.1002/jcp.22806 21503893

[B231] Yuan Y. Xue X. Ruo-Bing G. Xiu-Lan S. Gang H. (2012). Resveratrol enhances the antitumor effects of temozolomide in glioblastoma via ROS-dependent AMPK-TSC-mTOR signaling pathway. *CNS Neurosci. Ther.* 18 536–546. 10.1111/j.1755-5949.2012.00319.x 22530672 PMC6493617

[B232] Zeng W. Zhang W. Lu F. Gao L. Gao G. (2017). Resveratrol attenuates MPP^+^-induced mitochondrial dysfunction and cell apoptosis via AKT/GSK-3beta pathway in SN4741 cells. *Neurosci. Lett.* 637 50–56. 10.1016/j.neulet.2016.11.054 27894919

[B233] Zhang F. Shi J. S. Zhou H. Wilson B. Hong J. S. Gao H. M. (2010). Resveratrol protects dopamine neurons against lipopolysaccharide-induced neurotoxicity through its anti-inflammatory actions. *Mol. Pharmacol.* 78 466–477. 10.1124/mol.110.064535 20554604 PMC2939485

[B234] Zhang W. Fei Z. Zhen H. N. Zhang J. N. Zhang X. (2007). Resveratrol inhibits cell growth and induces apoptosis of rat C6 glioma cells. *J. Neurooncol.* 81 231–240. 10.1007/s11060-006-9226-x 17031560

[B235] Zhao H. F. Li N. Wang Q. Cheng X. J. Li X. M. Liu T. T. (2015). Resveratrol decreases the insoluble Aβ1-42 level in hippocampus and protects the integrity of the blood-brain barrier in AD rats. *Neuroscience* 310 641–649. 10.1016/j.neuroscience.2015.10.006 26454022

[B236] Zhou H. Liu X. Wu F. Zhang J. Wu Z. Yin H. (2016). Preparation, characterization, and antitumor evaluation of electrospun resveratrol loaded nanofibers. *J. Nanomater.* 2016:5918462 10.1155/2016/5918462

